# QSAR Modeling for Predicting IC_50_ and GI_50_ Values for Human Cell Lines Used in Toxicological Studies

**DOI:** 10.3390/ijms262412063

**Published:** 2025-12-15

**Authors:** Alexey A. Lagunin, Elena Y. Lisitsa, Anastasia V. Rudik, Sergey M. Ivanov, Alexander V. Dmitriev, Elena S. Muraviova, Dmitry A. Filimonov, Vladimir V. Poroikov

**Affiliations:** 1 Department of Bioinformatics, Institute of Biomedical Chemistry, 119121 Moscow, Russia; rudik_anastassia@mail.ru (A.V.R.); smivanov7@gmail.com (S.M.I.); a.v.dmitriev@mail.ru (A.V.D.); dmitry.filimonov@ibmc.msk.ru (D.A.F.); vladimir.poroikov@ibmc.msk.ru (V.V.P.); 2 Department of Bioinformatics, Pirogov Russian National Research Medical University, 117513 Moscow, Russia

**Keywords:** cytotoxicity, cell lines, in silico prediction, QSAR, preclinical toxicity assessment, GUSAR, CLC-Pred

## Abstract

Assessing cytotoxicity towards human cells is a critical step in preclinical drug development. In preclinical toxicology, human cell lines allow for the analysis of both general and organ-specific toxicity, thus, helping reduce development time and costs. Predicting cytotoxic IC_50_ and GI_50_ values facilitates the early evaluation of new pharmaceutical agents by assessing the possible therapeutic window. Ten non-tumor and 10 tumor cell lines commonly used in toxicology were selected to develop QSAR models using GUSAR software and ChEMBL data. GUSAR employs atom-centric electrotopological QNA and substructural MNA descriptors to encode molecular structure and utilizes the RBF–SCR algorithm to train QSAR models. The best-performing models (R^2^ > 0.5, RMSE < 0.8; mean R^2^ = 0.691, mean RMSE = 0.584) were selected using 5-fold cross-validation. These models were implemented in the freely available web application CLC-Pred 2.0 (Cell Line Cytotoxicity Predictor), initially developed for qualitative prediction of cytotoxicity in human cell lines.

## 1. Introduction

Preclinical toxicology studies are an important step in drug development that provides an early safety assessment prior to clinical trials. One of the key aspects in such studies is the assessment of cytotoxicity in cell lines, which allows the identification of the adverse effects of compounds on cell viability and the estimation of possible general, organ-specific, or tissue toxicity. Cell lines are populations of cells capable of long-term cultivation under artificial conditions. Human cell lines are diverse tools covering dozens of tissue types and hundreds of pathologies, making them indispensable in research. They are widely used to study disease mechanisms, evaluate drug-like compounds, and assess their toxicity. In preclinical toxicology studies, cell lines are used for the analysis of both general and organ-specific toxicity of substances. For example, HepG2 cells are used to assess hepatotoxicity [1], BEAS-2B cells are needed to describe the effects of substances on the respiratory tract [2], and HUVEC cells are employed to analyze vascular toxicity [3]. Due to their high reproducibility, standardization, and availability, cell lines are widely used in preclinical toxicology studies to assess the effects of chemical compounds on cell viability and function [4].

Modern cell lines are divided into tumor and non-tumor, as well as into transformed and normal cell lines. They are widely used to study the processes of cell division, apoptosis, genetic changes, and metabolic pathways, not to mention their role in the assessment of antitumor activity and toxicity of substances [5]. Currently, more than 6000 human cell lines have been registered in international databases, such as Cellosaurus (https://www.cellosaurus.org, accessed on 12 December 2025), covering a variety of tissues and organs, from skin and lungs to liver, kidneys, and the vascular system [6]. These include both tumor lines (e.g., HepG2, Caki-1) and normal (non-tumor) lines (e.g., NHDF, MRC-5, HUVEC). Such diversity allows the modeling of both systemic and organ-specific effects, including inflammation, metabolism, cellular senescence, and toxicity. The diversity of cell lines makes the selection of an experimental model more flexible and increases the reliability of results in preclinical studies. ATCC (American Type Culture Collection) includes over 500 human cell lines that may be used in toxicological studies (https://www.atcc.org/, accessed on 12 December 2025).

Today, there has been accumulated a large volume of experimental data on the cytotoxicity of compounds in relation to various cell lines. This enables the creation of predictive models, Quantitative Structure–Activity Relationship (QSAR) models among them. The development of QSAR models can significantly reduce both the time and cost of studies, providing toxicity predictions based on theoretical calculations when only the structural formula of a compound is known. The ChEMBL database [7] contains a significant body of information on experimental studies, including IC_50_ and GI_50_ values, making it an important and convenient resource for developing QSAR models [8]. PubChem also provides data on experimental studies of cytotoxicity for substances on different cell lines [9].

While traditional non-clinical testing relies predominantly on animal studies, regulatory agencies increasingly recognize computational approaches as complementary tools within integrated testing strategies. The International Council for Harmonization of Technical Requirements for Pharmaceuticals for Human Use (ICH M7(R2)) guideline represents a landmark achievement establishing the first internationally harmonized framework for the regulatory acceptance of QSAR predictions as an alternative to experimental testing in assessing bacterial mutagenicity of pharmaceutical impurities [10]. However, for organ-specific toxicity endpoints relevant to cell line-based assessments, no equivalent regulatory frameworks have been developed so far to formally endorse QSAR models as a replacement for in vivo studies. The OECD Guidance Document on QSAR Validation previously established five essential principles for model validation: defined endpoint, unambiguous algorithm, defined applicability domain, appropriate validation metrics, and mechanistic interpretation [11]. The recent OECD (Q)SAR Assessment Framework extends these principles with a systematic methodology for the regulatory assessment within defined contexts of use [12]. Major regulatory initiatives explicitly support New Approach Methodologies (NAMs) development. The FDA’s Predictive Toxicology Roadmap (2017) emphasizes the “context of use” as the foundation for qualification and regulatory acceptance [13]. The FDA/CDER 2020 commentary [14] acknowledges that improvements increasing clinical outcome predictivity are encouraged and needed, explicitly recognizing QSAR as NAMs. The EMA NAMs Horizon Scanning Report [15] confirms that while no NAMs are currently qualified for regulatory use, such methodologies are “advancing to technology readiness levels sufficient for initial engagement with regulators.” The trajectory established by ICH M7(R2) for mutagenicity prediction provides a precedent suggesting that as experience accumulates with organ-specific toxicity QSAR models and validation datasets expand, similar regulatory frameworks may emerge for cell-based cytotoxicity predictions. The models presented in our study align with the 3Rs principles (Replacement, Reduction, Refinement) and OECD validation principles, providing hazard identification, compound prioritization, and mechanistic investigation in drug development.

Recently, Feitosa with co-authors developed the Cyto-Safe web application with SAR models for predicting cytotoxicity of substances on two cell lines, 3T3 and HEK 293 [16]. Earlier, we developed freely available web applications CLC-Pred [17] and CLC-Pred 2.0 [18] with SAR models that made qualitative predictions of compound toxicity against hundreds of tumor and non-tumor human cell lines based on ChEMBL and PubChem data. Such web applications provide a general idea of cytotoxic potential against cell lines but do not offer quantitative assessments that are important when comparing the activity level. The estimation of IC50 and GI50 values is crucial for the calculation of therapeutic interval and dosage of substances in preclinical and clinical studies and can serve as a characteristic used to select the most promising drug candidates.

Verma and Hansch performed one of the earliest QSAR models of podophyllotoxin derivatives for four cancer cell lines using physicochemical descriptors. The resulting models demonstrated a high degree of agreement with the experimental data (R^2^ from 0.960 to 0.836) and a good predictive ability (Q^2^ from 0.911 to 0.705) [19]. Q^2^ is the cross-validated R^2^, determined by a leave-one-out procedure on the training set. Earlier, we also demonstrated the development and implementation in BC CLC Pred web-application of QSAR models to predict IC_50_ and GI_50_ values for compounds tested on nine human breast cancer cell lines with a reasonable accuracy of prediction (mean R^2^ and RMSE values calculated by 5-fold cross-validation were 0.599 and 0.679, respectively) [8].

As most scientists lack the opportunity to test substance cytotoxicity on panels of human cell-lines [8], the development of a freely available in silico tool to predict quantitative values of cytotoxicity against different non-tumor and tumor human cell lines is in high demand. In this study we created high quality QSAR models by GUSAR software (version 2014) [8,20,21] for dozens of human cell lines, which have been implemented in the CLC-Pred 2.0 web application (https://way2drug.com/clc-pred/, accessed on 12 December 2025) [18].

## 2. Results

### 2.1. Data Analysis

Starting with ATCC data on human cell lines designated for toxicological studies, we analyzed the ChEMBL database to identify cell lines with sufficient amount of experimental data (number of unique compounds > 100) with definitive quantitative IC_50_ or GI_50_ values. As a result, 17 non-tumor and 15 tumor human cell lines were selected. The experimental data and structures of compounds were extracted from ChEMBL (v.33). IC_50_ and GI_50_ values were converted to pIC_50_ and pGI_50_ values, respectively. For duplicate chemical structures, the corresponding data were consolidated by calculating the median value. Records with inappropriate structures, such as mixtures, charged structures, inorganic compounds, or compounds with a molecular mass greater than 1250 Daltons, were removed. As a result, training sets were created for each selected human cell line. The characteristics of training sets for non-tumor and tumor cell lines are given in Table 1 and Table 2, respectively. Table 1 shows that non-tumor cell lines covering a wide range of tissues were selected: skin (BJ, HaCaT, HFF, NHDF), gastrointestinal tract (CCD-18Co, GES1), kidney (HEK-293, HEK-293T), lung (BEAS-2B, HFL1, MRC-5, WI-38), blood vessels (HUVEC, HMEC-1), breast (MCF-10A), blood (PBMC), and retinal pigment epithelial cells (TERT-RPE1).

Seven cell lines (BJ, CCD-18Co, HFF, HFL1, MRC-5, NHDF, and WI-38) are fibroblasts, seven (BEAS-2B, GES1, HEK-293T, HEK293, HUVEC, MCF-10A, TERT-RPE1) are epithelial cells, two (HMEC-1, HUVEC) represent endothelial cells, and one (HaCaT) keratinocytes. Most of the datasets contained IC_50_ values, whereas datasets with GI_50_ values were created only for HUVEC and MRC-5 cell lines. For most cell lines, the mean pIC_50_ and pGI_50_ values are around 5 (excluding PBMC and HUVEC that had values close to 6), which is the generally accepted threshold between active and inactive compounds (pIC_50_ and pGI_50_ values of 5 correspond to a concentration of 10 µM). This indicates that the datasets contain sufficient numbers of both active and inactive compounds, and QSAR models created using these data will enable an effective discrimination between active and inactive compounds. QSAR models typically tend to bias their predictions toward the mean [22], so it is important that this value lies near the generally accepted boundary between active and inactive compounds. At the same time, a wide range of values is observed across the datasets. It is always above 3 logarithmic units, which is also one of the indicators of the potential to create good QSAR models. Thus, the formal parameters of the data in the training sets indicate that there were no obstacles in creating accurate QSAR models using these data.

Table 2 shows that the selected tumor cell lines cover a wide range of tissues, including skin (A-375), lung (A-549, Calu-3), colon (Caco-2, COLO 205, HCT-8, SW-620), liver (HepG2), kidney (Caki-1), blood (THP-1), as well as nervous (SH-SY5Y) and lymphoid (U-937) tissues, allowing for the modeling of organ-specific toxic effects. Nine tumor cell lines from Table 2 belong to carcinoma (A-431, Caki-1, SCC-25) and adenocarcinoma (A-549, Caco-2, Calu-3, COLO 205, HCT-8, SW-620). The list of tumor cell lines includes also melanoma (A-375), neuroblastoma (SH-SY5Y), acute monocytic leukemia (THP-1), and lymphoma (U-937).

Table 2 shows the average values of the studied compounds’ activity against tumor cell lines, along with their ranges in pIC_50_ and pGI_50_ values. These data, as for non-tumor cells, show that for most cell lines, the average values of pIC_50_ and pGI_50_ are around 5 (excluding HCT-8, COLO 205, and THP-1 for pIC_50_ together with Caki-1, COLO 205, and SW-620 approaching 6). Moreover, the range of values in the datasets is even greater than that for non-tumor cells, exceeding 5 logarithmic units everywhere. Thus, the formal parameters of the data in the training sets for tumor cell lines indicate that there were also no obstacles in creating accurate QSAR models using these data.

### 2.2. QSAR Modeling

QSAR models were made by GUSAR software based on the created training sets that are described in Table 1 and Table 2 (see Section 4). The threshold Q^2^ > 0.5 was used to estimate the quality of the created QSAR models for further 5-fold cross-validation (5-fold CV). The predictive performance, Q^2^, was calculated using the leave-one-out cross-validation of the training data during the creation of QSAR models (the scatter plots of predicted vs. known experimental data given during this validation are represented in the Supplement (Figures S1–S35)). All QSAR models used to predict pIC_50_ values for 17 non-tumor cell lines met the threshold (Table 3). The accuracy of the QSAR model for the prediction of pGI50 values for HUVEC was also high enough (Table 3) excluding the one for MRC-5. The results of the 5-fold cross-validation of QSAR models for non-tumor cell lines (when 20% of compounds from the initial datasets were used as the validation set and 80% became the training set in each iteration) are presented in Table 3.

Table 3 shows that despite the reasonable accuracy of prediction given in full training sets, stricter 5-fold CV revealed seven weak QSAR models predicting pIC_50_ values (with R^2^_5-fold CV_ value less 0.5) for BEAS-2B, BJ, CCD-18Co, HFF, HFL1, HMEC-1, and NHDF cell lines. As a result, 10 QSAR models predicted pIC_50_ values and one QSAR model predicted a pGI_50_ value (with R^2^_5-fold CV_ value over 0.5 and RMSE_5-fold CV_ less than 1) which may be considered acceptable to be employed in toxicological assessment of new compounds. The most accurate QSAR models were obtained for predicting pIC_50_ values in HaCaT, HEK-293T, and MCF-10A cell lines with R^2^_5-fold CV_ more than 0.8 and RMSE_5-fold CV_ less than 0.5. The average R^2^_5-fold CV_ and RMSE_5-fold CV_ of these models achieved the 0.695 and 0.569 values, respectively. The negative R^2^_5-fold CV_ values for HFL1 and NHDF cell lines could be obtained because a significant number of the highly active compounds in the test set had been predicted to be less active.

QSAR modeling based on the training sets related to tumor cell lines (mentioned in Table 2) displays different patterns (Table 4). The reasonable QSAR models predicted pIC_50_ values were created only for 12 tumor cell lines (shown in Table 4) of 15 (Q^2^ > 0.5). At that time, QSAR models predicted pGI50 values for six of eight tumor cell lines revealing Q^2^ exceeding 0.5.

Table 4 shows that the 5-fold CV procedure (when 20% of compounds from the initial datasets were used as the validation set and 80% became the training set in each iteration) revealed that only eight QSAR models predicted pIC_50_ values (A-431, Caco-2, COLO 205, HCT-8, HepG2, SW-620, THP-1 and U-937) and predicted pGI_50_ values (A-431, THP-1 and U-937) with a reasonable accuracy of prediction (R^2^_5-fold CV_ value > 0.5 and RMSE_5-fold CV_ < 1). They may also be considered acceptable to be used in cytotoxic assessment of new compounds. The most accurate QSAR models were for the prediction of pGI_50_ values in THP-1 and U-937 cell lines with R^2^_5-fold CV_ over 0.8 and RMSE_5-fold CV_ less 0.5. The average R^2^_5-fold CV_ and RMSE_5-fold CV_ of these models have approximately the same values as for non-tumor cell lines: 0.686 and 0.599, respectively. The biological description of the cell lines from Table 3 and Table 4 is given in Table 5.

Table 5 shows the variety of cell lines used in various toxicology studies to assess both general and organ-specific toxicity. By using these cell lines, one can evaluate such types of toxicity as hepatotoxicity, nephrotoxicity, immunotoxicity, dermatotoxicity, gastrointestinal toxicity, and lung toxicity. Unfortunately, cell lines associated with important types of toxicity such as cardiotoxicity, bone marrow, and neurotoxicity are not included in the list of cell lines for which it was possible to create acceptable QSAR models.

### 2.3. Web Application

**Figure 1:**
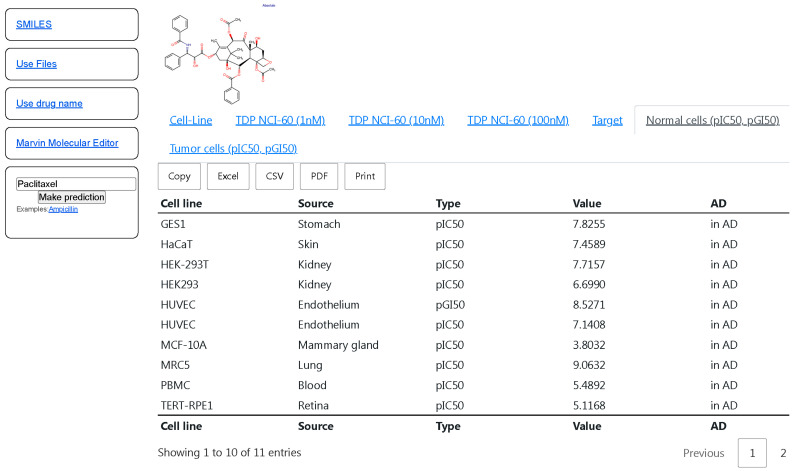
The interface of CLC-Pred with the prediction results of pIC_50_, pGI_50_ values on non-tumor cell lines for paclitaxel. The titles of the columns mean the following: “Cell line”—name of cell lines; “Type”—pIC50 or pGI50; “Value”—predicted pIC_50_, pGI_50_ values; “AD”—Applicability Domain (“In AD”—in the Applicability Domain, “out of AD”—out of Applicability Domain.

The selected QSAR models (with R^2^_5-fold CV_ value more than 0.5 and RMSE_5-fold CV_ less than 1) based on the results of the 5-fold CV procedure were implemented in CLC-Pred 2.0 (https://way2drug.com/clc-pred/, accessed on 12 December 2025) [18]. This web-application provides users with a possibility to submit the drug name, SMILES strings [62], upload a file in the ‘MOL’ format [63], and draw or insert the structural formula in Marvin JS applet as input data for the prediction. The prediction is output in the form of tables with pIC_50_ and pGI_50_ values and the estimation of applicability domain or each cell line is represented in two tabs—‘Normal cells (pIC50, pGI50)’ and ‘Tumor cells (pIC50, pGI50)’ for non-tumor and tumor cell lines, respectively (Figure 1). By clicking on the title of the column, users can sort the prediction results based on the data from the column. The prediction results may be copied to the clipboard or exported to CSV, Excel, or PDF files, and also printed.

Figure 1 displays the high predicted pIC_50_ values (more 6) of cytotoxicity for the most non-tumor cell lines for anti-tumor agent paclitaxel. Although paclitaxel is widely used in the treatment of different types of solid tumors, such as breast, colon, prostate, non-small-cell lung, and ovarian cancers, it is also known for its multiorgan toxicity [64]. Therefore, the presented predicted results for paclitaxel reflect its potential to cause toxic effects observed in the clinic.

## 3. Discussion

The ability to quantitatively predict IC_50_ and GI_50_ values for sets of non-tumor and tumor cell lines provides an opportunity to select the most promising drug candidates at the early stages of drug development. This can be best demonstrated using anticancer drugs comparing paclitaxel (Taxol), that has a broad spectrum of cytotoxic effect on both non-tumor and tumor cells, with the targeted anticancer drugs trametinib and dabrafenib. Trametinib, which is an MEK inhibitor, and dabrafenib, which is a BRAF inhibitor, are used to treat solid tumors, lymphomas, or multiple myeloma with BRAF V600E driver mutation [65]. The prediction results of pIC_50_ and pGI_50_ values made by the created QSAR models are presented in Table 6.

Table 6 shows that the prediction results for paclitaxel include high values of pIC_50_ and pGI_50_ (more 7) for many non-tumor and tumor cell lines. It correlates with the knowledge on general toxicity [66] and organ-specific toxicity of paclitaxel: gastrointestinal toxicity (esophagus, stomach, small intestine) [67], hepatotoxicity [68], endothelium toxicity [69], lung toxicity [70], skin toxicity [71], and immunotoxicity [72,73] associated with its mechanism of action—polymerized microtubule accumulation and mitotic arrest. At the same time, the high values of pIC_50_ and pGI_50_ for some tumor cell lines reflect several main fields of therapeutic applications of paclitaxel: melanoma, lung, and esophageal cancer [74]. Despite the high predicted cytotoxicity values for non-tumor cell lines averaging 6.889, the average predicted cytotoxicity value for tumor cells is approximately four times higher at 7.502 (7.502 − 6.889 = 0.613 in logarithmic scale). The log-transformed value of 0.613 corresponds to a decimal value of 4.1. It means that paclitaxel is, on average, more cytotoxic against tumor cells than non-tumor ones. This reflects the existence of a reasonable therapeutic window (TI—Therapeutic Index = 4.1) for its clinical use. Hertz with co-authors also claimed that paclitaxel is similar to many anticancer agents in having a relatively narrow therapeutic window [75].

The prediction results for trametinib and dabrafenib indicate that they may be less toxic than paclitaxel. Their mean predicted cytotoxicity values for non-tumor cell lines were 5.604 and 5.328, respectively. Trametinib and dabrafenib are targeted therapy drugs, and they are considered less toxic than chemotherapy drugs such paclitaxel [76,77]. The low predicted values of cytotoxicity for trametinib and dabrafenib on non-tumor cell lines correlate with the absence of dangerous organ-specific toxic effects of these drugs which may be related to cytotoxicity in these cell lines [78]. Nevertheless, the prediction results with cytotoxic values higher than average ones in non-tumor cell lines may be associated with some types of adverse drug reactions of these drugs. Skin-related toxic effects, hypertension, and diarrhea caused by trametinib [79] may be associated with the prediction of high cytotoxic action on skin A-431, endothelial HUVEC [80], and colon (COLO205, HCT-8, SW-620) cell lines, respectively. Dabrafenib also reveals cutaneous adverse reactions and diarrhea [81] that may be associated with the prediction results for skin A-431 and colon (COLO205, HCT-8) cell lines, respectively. Moreover, Table 6 includes high predicted values of cytotoxicity for blood cells. While less frequent and severe than other side effects like pyrexia and skin toxicity, hematologic adverse reactions such as anemia, leukopenia, and neutropenia for dabrafenib [82] and anemia for trametinib [83] have also been reported. The mean predicted cytotoxicity values of trametinib and dabrafenib for tumor cell lines were 6.241 and 6.119, respectively. These values indicate that the cytotoxicity of trametinib and dabrafenib on tumor cell lines is higher than on non-tumor cell lines. The difference between the average predicted cytotoxic values of these drugs for non-tumor and tumor cell lines also displays the existence of a therapeutic interval. It is higher for dabrafenib (6.119 − 5.328 = 0.791 in logarithmic scale) than for trametinib (6.241 − 5.604 = 0.637 in logarithmic scale). The log-transformed values of 0.791 and 0.637 correspond to decimal values of 6.2 and 4.3, respectively, reflecting the estimation of therapeutic indexes of dabrafenib and trametinib. This correlates with the data on the narrow therapeutic window for these drugs [84,85]. There are no strict criteria for TI. TI ≥ 10 is considered preferable for selecting universal drugs and TI > 5 is a criterion for considering a drug candidate for further preclinical study [86]. Nevertheless, a low TI (<2), known as a narrow therapeutic index drug, is also used for some severe diseases [87].

This illustration along with the comparison of prediction results of pIC_50_ and pGI_50_ values for paclitaxel, trametinib, and dabrafenib show the usefulness of the created QSAR models for the estimation of possible toxicity of drug-candidates and their potential in terms of the presence of a therapeutic window. In the future, when data on new drugs become available, it would be advisable to conduct similar studies on a larger external set to more accurately assess the possibility of using the created QSAR models to determine the therapeutic interval and toxicity of novel pharmaceutical agents.

Regarding the QSAR models themselves, the resulting model analysis revealed that the quality of QSAR predictions depends significantly on the cell line type. In particular, for some lines, models with high R^2^_5-fold CV_ (>0.6) and RMSE_5-fold CV_ (<0.8) values were obtained, indicating high accuracy and reproducibility of the predictions. This is typical, for example, of the HaCaT, GES-1, and HUVEC cell lines that have proven to be successful in cytotoxicology studies, and which exhibit a stable phenotype in vitro.

Organ-specific characteristics also influence the cytotoxic response. For example, cell lines mimicking barrier tissues (skin-HaCaT, intestine-Caco-2, respiratory tract-Calu-3) demonstrated specific sensitivities to certain classes of compounds, which is important to consider when interpreting the results. Endothelial cells (HUVEC) also demonstrated highly reproducible cytotoxic effects, making them useful for assessing angiotoxicity. Liver cell lines (HepG2) have metabolic activity allowing for the impact of compound biotransformation to be considered; however, their predictive accuracy may vary depending on the specific compounds tested.

The comparison of non-tumor and tumor cell lines revealed some trends: in general, models built on non-tumor cells are characterized by higher stability; however, they are sometimes inferior in accuracy to models based on tumor lines, especially if the latter have a pronounced mitotic potential (e.g., Caki-1, A549). This is because tumor lines often have a simplified response to chemical exposure due to impaired regulation of apoptosis and the cell cycle, which facilitates the detection of cytotoxic effects. Non-tumor cell lines, in contrast, are closer to physiological norms, making their results more relevant for assessing potential toxicity in the body, but this requires a more precise modeling approach. Due to the narrow therapeutic index of certain drugs, it is crucial to have highly accurate QSAR models. However, not all QSAR models achieve high predictive performance. To address this limitation and enhance the reliability of the therapeutic index estimates, the following strategies can be employed:

1. Prioritizing high-accuracy QSAR models: Selecting prediction results from models with the highest accuracy, such as models for predicting IC_50_ values in non-tumor cell lines (HaCaT, HEK-293T, MCF-10A) as well as in tumor cell lines (COLO 205, HCT-8), and GI_50_ values in tumor cell lines (THP-1, U-937)
2. Leveraging models with medium accuracy but large training sets: Consider QSAR models with moderate predictive performance if they are built on extensive training datasets, e.g., for non-tumor cell lines (HEK-293, MRC-5) and tumor cell lines (A-431, THP-1, U-937). Such models typically cover a broader chemical space and exhibit greater robustness due to the diversity of the training data
3. Excluding predictions beyond the applicability domain (AD): Avoid using predictions marked as “out of AD” that can be identified in the following cases.
  - the query structure exhibits less than 70 % similarity to any compound in the training set
  - the predicted value deviates from the values of the three most similar compounds in the training set by more than the RMSE values of a model.

In summary, although the accuracy of individual QSAR models may vary, their combined use can help to overcome the limitations of any one model and provide a more reliable estimation of the therapeutic index.

The results show that the quality of QSAR models depends on both the amount of experimental data and the cell line type. Immune system cell lines (THP-1, U-937) demonstrated better predictive power, possibly due to a more robust biological response and lower heterogeneity. However, for the lines with high biological heterogeneity (e.g., COLO 205 (pGI_50_) and A549), the models were less accurate.

Therefore, when developing and using QSAR models for cytotoxicity prediction, it is important to consider both the biological nature of the cell line and the specifics of its application. Combining tumor and non-tumor models provides a more comprehensive understanding of a compound’s toxicity profile and increases the reliability of in silico assessments.

Increasing the number of experimental IC_50_ and GI_50_ values for both active and inactive compounds will lead to the creation of new efficient and reliable QSAR models for a wide range of cell lines and will help to efficiently discover new promising drugs at an early stage of their development. The current implementation of GUSAR is based on atomic neighborhood descriptors QNA and MNA, as well as several physico-chemical descriptors. It also uses the original SCR (Self-Consistent Regression) and RBF–SCR (Radial Basis Function network using SCR) algorithms. The use of additional molecular descriptors and machine learning algorithms to improve the accuracy and predictivity of QSAR models for cytotoxicity prediction may be the subject of discussion. However, as the widespread use of QSAR has demonstrated, the current accuracy of the obtained models is primarily limited by the quantity and accuracy of the experimental data used for training and testing [20,88,89].

## 4. Materials and Methods

### 4.1. Data

ATCC (available in October 2025) was used for selection of human cell lines related to toxicology studies. The query ‘Toxicology’ and filters ‘Human cells’ in ‘Product category’ and ‘Toxicology’ in ‘Product application’ of query sections were used, and more than 500 human cell lines were selected. The records were analyzed by the number of ‘Product Citations’, and human cell lines with the highest number of citations (>1000) were selected. The information on human non-tumor cell lines with a significant number of cytotoxic compounds from our previous study [18] was also used for the creation of a list of human cell lines for further investigation. As a result, a list of 33 human non-tumor cell lines and 24 tumor cell lines was created.

The ChEMBL database (v. 33) was used as a source of data on structures of compounds and experimental IC_50_ and GI_50_ values. IC_50_ (the inhibitor concentration that causes a 50% reduction in target activity) is one of the most common parameters for assessing cytotoxicity (in this case, the target is a cell line). GI_50_ (the concentration that results in 50% inhibition of cell growth) is used in antiproliferative activity studies. These parameters characterize the interaction of a compound with a biological target and allow for comparison of their toxicity and efficacy. A search of compounds with exact IC_50_ or GI_50_ values in nM was performed for each selected cell line. IC_50_ or GI_50_ values were converted to logarithmic views of pIC_50_ (−log10(IC_50_/10^6^) and pGI_50_ (−log10(GI_50_/10^6^). Duplicate records with the same structures were consolidated by median pIC_50_ or pGI_50_ calculation.

### 4.2. GUSAR Software

QSAR models were made by GUSAR software [8,20,21], which uses Quantitative Neighborhoods of Atoms (QNA) [20] and Multilevel Neighborhoods of Atoms (MNA) descriptors [88] to describe structural formulae of compounds and the SCR–RBF algorithm [89] to build quantitative structure–activity relationships. Whole-molecule descriptors including topological length, topological volume, lipophilicity, number of positive charges, number of negative charges, number of hydrogen bond acceptors, the number of hydrogen bond donors, the number of aromatic atoms, molecular weight, and the number of halogen atoms were also used in GUSAR [21]. The applicability domain estimation is also implemented in GUSAR. It is calculated during the prediction of query compounds. An estimation of the applicability domain (AD) is based on its simultaneous calculation by three methods (similarity, leverage, and accuracy assessment). These methods of AD calculation were used by default [21]. It is enough for one of the methods to signal that the structure is outside the AD for this inscription to appear in the prediction results. In this case, the prediction results should be treated more critically.

For each training set, 320 QSAR models were created by GUSAR with a modified calculation of descriptors and regression coefficients. Based on the internal leave-many-out cross-validation (LMO CV, when 20% from the training set were used as an internal test, repeated 5 times), single QSAR models with the reasonable values (R^2^ > 0.5, Q^2^ > 0.5 for the training set and R^2^ of internal LMO CV > 0.5) were selected for the creation of the final consensus QSAR models for each training set.

R^2^, coefficient of determination and Root Mean Square Error (RMSE) were calculated as follows:

(1) $$R^{2}=1-\frac{\sum (y_{\exp}-y_{pred}{)}^{2}}{\sum (y_{\exp}-y_{mean}{)}^{2}},$$

(2) $$\mathrm{RMSE}=\sqrt{\frac{\sum (y_{\exp}-y_{pred}{)}^{2}}{n}}$$

where *y_exp_*—experimental value, *y_pred_*—predicted value, *y_mean_*—average value of experimental values in a training set, and *n* is the number of objects. The R^2^ values represent the relationship between the predicted and the observed values of the measured biological activity [21] and R^2^ values closer to 1 indicate successful predictions. R^2^ of the internal LMO CV was calculated as R^2^ (Equation (1)). Q^2^ is a cross-validated R^2^ calculated during the leave-one-out cross-validation procedure using the data from a training set.

## Figures and Tables

**Table 1:** General characteristics of non-tumor cell lines and corresponding datasets.

Cell Line	ATCC Accession Number	Description	Tissue	Interval of pIC_50_	Mean pIC_50_	Interval of pGI_50_	Mean pGI_50_
BEAS-2B	CRL-3588	Bronchial epithelial cells	Lung	2.6–8.1	4.9		
BJ	CRL-2522	Foreskin fibroblast	Skin	1.3–9.7	5.1		
CCD-18Co	CRL-1459	Fibroblasts	Colon	3.9–7.4	5.5		
GES1	-	Gastric epithelial cells	Stomach	3.3–8.1	4.8		
HaCaT	-	Epidermal keratinocytes	Skin	3.2–10.5	5.3		
HEK293	CRL-1573	Embryonic kidney epithelial cells	Kidney	1.2–10.5	5.1		
HEK-293T	CRL-11268	Renal epithelium	Kidney	2.2–9.1	4.8		
HFF	SCRC-1041	Foreskin fibroblast	Skin	3.6–9.3	5.1		
HFL1	CCL-153	Human fetal lung fibroblast	Lung	3.7–8.0	5.5		
HMEC-1	CRL-3243	Dermal microvascular endothelial cell	Skin	3.6–8.6	5.6		
HUVEC	PCS-100-013	Umbilical vein endothelial cells	Endothelium	1.7–12.3	5.8	3.8–10.2	5.5
MCF-10A	CRL-10317	Epithelial cells of the mammary gland	Mammary gland	1.8–9.6	4.9		
MRC-5	CCL-171	Embryonic lung fibroblasts	Lung	1.6–9.3	4.8	3.3–7.4	5.0
NHDF	PCS-201-012	Fibroblast	Skin	3.0–8.8	4.9		
PBMC	PCS-800-011	Peripheral blood mononuclear cells	Blood	2.3–11.2	6.1		
TERT-RPE1	CRL-4000	Pigmented retinal epithelial cells	Retina	4.1–6.9	5.0		
WI-38	CCL-75	Embryonic lung fibroblast	Lung	2.7–8.0	5.0		

**Table 2:** General characteristics of tumor cell lines and corresponding datasets.

Cell Line	ATCC Accession Number	Description	Tissue	Interval of pIC_50_	Mean pIC_50_	Interval of pGI_50_	Mean pGI_50_
A-375	CRL-1619	Melanoma	Skin	0.4–11.0	5.7		
A-431	CRL-1555	Epidermoid carcinoma	Epithelium	1.6–11.2	5.6	2.9–9.9	5.4
A-549	CCL-185	Adenocarcinoma	Lung	0.03–12.0	5.4	2.9–13.6	5.6
Caco-2	HTB-37	Adenocarcinoma	Colon	2.1–8.5	5.0		
Caki-1	HTB-46	Carcinoma	Kidney	1.5–9.5	5.6	4.0–13.8	5.8
Calu-1	HTB-54	Epidermoid carcinoma	Lung	3.0–9.4	5.4		
Calu-3	HTB-55	Adenocarcinoma	Lung	1.7–8.8	4.9		
COLO 205	CCL-222	Adenocarcinoma	Colon	2.0–11.5	5.8	4.0–13.8	5.8
HCT-8	CCL-244	Ileocecal adenocarcinoma	Colon	4.0–9.2	6.0		
HepG2	HB-8065	Hepatoblastoma	Liver	0.5–11.0	5.1	2.4–9.1	5.3
SCC-25	CRL-1628	Squamous cell carcinoma	Tongue	1.9–9.2	5.2		
SH-SY5Y	CRL-2266	Neuroblastoma	Nervous	2.6–9.7	5.3		
SW-620	CCL-227	Adenocarcinoma	Colon	0.5–10.7	5.5	4.0–14.0	5.8
THP-1	TIB-202	Acute monocytic leukemia	Blood	2.2–12.0	5.8	4.0–9.0	5.4
U-937	CRL-1593.2	Histiocytic lymphoma	Lymphoid	3.0–11.0	5.6	4.2–10.4	6.3

**Table 3:** Accuracy characteristics for QSAR models for non-tumor cell lines. The thresholds for selection of reasonable QSAR models are R^2^_5-fold CV_ > 0.5 and RMSE_5-fold CV_ < 1.

Cell Line	Tissue	N *	R^2^_5-Fold CV_	RMSE_5-Fold CV_
*pIC_50_*				
BEAS-2B	Lung	129	0.497	0.550
BJ	Skin	328	0.340	0.854
CCD-18Co	Colon	311	0.201	0.395
GES1	Stomach	192	0.610	0.503
HaCaT	Skin	730	0.872	0.371
HEK-293	Kidney	2705	0.695	0.607
HEK-293T	Kidney	588	0.826	0.409
HFF	Skin	454	0.207	0.873
HFL1	Lung	341	−0.298	0.663
HMEC-1	Skin	152	0.473	1.029
HUVEC	Endothelium	2254	0.553	0.920
MCF-10A	Mammary gland	514	0.818	0.357
MRC5	Lung	1523	0.688	0.493
NHDF	Skin	234	−0.249	0.581
PBMC	Blood	1885	0.540	0.969
TERT-RPE1	Retina	96	0.623	0.407
WI-38	Lung	829	0.786	0.508
*pGI_50_*				
HUVEC	Endothelium	239	0.631	0.711

* N—number of compounds in the training set.

**Table 4:** Accuracy characteristics for QSAR models for tumor cell lines. The thresholds for selection of reasonable QSAR models are R^2^_5-fold CV_ > 0.5 and RMSE_5-fold CV_ < 1.

Cell Line	Tissue	N *	R^2^_5-Fold CV_	RMSE_5-Fold CV_
*pIC_50_*				
A-375	Skin	3020	0.430	0.881
A-431	Epithelium	2496	0.695	0.610
Caco-2	Colon	825	0.577	0.533
Caki-1	Kidney	323	NA **	NA **
Calu-1	Lung	177	0.496	0.811
COLO 205	Colon	1334	0.700	0.639
HCT-8	Colon	980	0.737	0.530
HepG2	Liver	16982	0.554	0.762
SH-SY5Y	Nervous	826	0.471	0.782
SW-620	Colon	1918	0.566	0.698
THP-1	Blood	2629	0.684	0.803
U-937	Lymphoid	1503	0.669	0.682
*pGI_50_*				
A-431	Epithelium	373	0.647	0.550
COLO 205	Colon	1319	−0.283	1.052
HepG2	Liver	653	0.487	0.671
THP-1	Blood	114	0.851	0.384
U-937	Lymphoid	147	0.882	0.442

* N—number of compounds in the training set; ** NA—Not available (QSAR modeling during 5-fold cross-validation failed).

**Table 5:** Description of human non-tumor and tumor cell lines related with the selected QSAR models.

Cell Line	Detailed Description	Toxicity Study
*Non-tumor cell lines*		
GES1	This line is derived from normal gastric epithelial cells. Transfection of miR-23a microRNA enhanced cell proliferation, invasion, and colony formation, making the line valuable for studying oncogenesis. Expression of miR-23a enhances the aggressive properties of cells, demonstrating their potential for evaluating compounds that affect genes or microRNAs [23].	Gastrotoxicity [24]
HaCaT	These are immortalized human skin cells used to study skin diseases. They help understand how immune proteins (IFNγ, IL-4, IL-17A, IL-22) influence skin cell function [25].	Skin toxicity [26]
HEK293, HEK-293T	Human embryonic kidney cells are widely used for the production of recombinant proteins and viral vectors. HEK-293 was produced by transforming cells with adenovirus, and HEK-293T is a modified version expressing SV40 antigen, which increases transfection efficiency. The lines are characterized by rapid growth, high transfection efficiency and the possibility to be cultivated in suspension [27,28].	Nephrotoxicity [29,30]
HUVEC	Human Umbilical Vein Endothelial Cells are endothelial cells isolated from the human umbilical vein. These cells are used to model vascular permeability, study inflammatory mechanisms, and test angiogenic agents. The advantages of HUVEC include the ease of isolation, high reproducibility, and the ability to be used in co-cultures with other cell lines. Limitations include short culture time due to the natural characteristics of primary cells and donor-specific variability [3].	Different toxicological studies [31]
MCF-10A	These are human mammary gland epithelial cells used to study the regulation of cell growth and signaling. These cells possess characteristics of normal epithelium, including the ability to differentiate and form organelles. MCF-10A cells exhibit growth stability, high sensitivity to external stimuli, and the possibility of genetic modification [32].	Different toxicological studies [33]
MRC-5	These are human lung fibroblasts obtained from embryonic tissue. These cells demonstrate high sensitivity to coronavirus infection and are used to evaluate the effectiveness of antiviral compounds [34].	Lung toxicity [35]
PBMC	PBMCs are Peripheral Blood Mononuclear Cells, a heterogeneous population of immune cells including T lymphocytes, B lymphocytes, natural killer (NK) cells, and monocytes isolated from whole blood using gradient centrifugation. PBMCs are widely used in preclinical and clinical studies to analyze immune responses, test drug compounds, and study inflammatory mechanisms [36].	Immunotoxicity [37]
TERT-RPE1	This is a human retinal pigment epithelial cell line immortalized by expressing telomerase reverse transcriptase (hTERT). TERT-RPE1 is widely used in molecular biology and toxicology to study cytoskeletal dynamics, signaling pathways, and genetic modifications [38].	Comparative toxicological studies [39,40]
WI-38	This is a cell line of diploid human lung fibroblasts obtained from the lung tissue of a 3-month-old embryo. WI-38 is widely used in research on aging, tissue regeneration, and virology. The advantages of this line include a stable karyotype, reproducible results, and safety in biomedical applications [41].	Comparative toxicological studies [42]
*Tumor cell lines*		
A-431	This is a human epidermoid carcinoma cell line characterized by high expression of epidermal growth factor receptors (EGFR). This cell line is widely used to study the mechanisms of action of antitumor drugs aimed at inhibiting the EGFR signaling pathway, as well as in dermatotoxicological studies [43].	Skin toxicity [44]
Caco-2	This is a human colorectal adenocarcinoma cell line that, upon reaching confluence, differentiates into cells similar in morphology and functions to small intestinal enterocytes. Caco-2 is the gold standard in vitro assay for modeling intestinal epithelium and is widely used to assess the absorption, transport, and cytotoxicity of compounds acting through the gastrointestinal tract [45].	Intestinal toxicity [46]
COLO 205	This is a cell line derived from the ascitic fluid of a patient with metastatic colorectal adenocarcinoma. The cells grow both in suspension and in an adherent form, which makes them a convenient model for studying tumor growth dynamics. COLO 205 is used to evaluate the antitumor activity of compounds [47,48].	Comparative toxicological studies of anticancer drugs [49]
HCT-8	This is an epithelial cell line derived from human colon adenocarcinoma. The cell model is widely used in gastroenterology and oncology research, as well as in assessing the cytotoxicity of anticancer drugs [50]	Intestinal toxicity [51]
HepG2	Hepatocellular Carcinoma G2 (HepG2) cell line is one of the most widely used cell lines for studying liver metabolism, toxicology, and oncology. Although HepG2 has been widely used as a liver model, its genetic and metabolic characteristics limit its predictive value in studies of hepatocellular carcinoma and liver metabolism [1]	Hepatotoxicity [52]
SW-620	This is a human colorectal adenocarcinoma cell line derived from a lymph node metastasis in a patient with advanced colon cancer. The line is actively used to study late stages of colorectal cancer, invasion, metastasis, and resistance to antitumor drugs [53].	Comparative cytotoxic studies of anticancer drugs [54]
THP-1	This is a cell line derived from the peripheral blood of a patient with acute monoblastic leukemia. It is actively used as a model for studying the innate immune response, inflammation, and the cytotoxicity of immunomodulatory and anti-inflammatory compounds [55].	Long-term safety assessment [56], Skin sensitization [57], Immunotoxicity [58]
U-937	This is a monocytoid cell line derived from human lymphoma that is often used as a model of monocytes and macrophages in immunological and inflammatory studies [59].	Skin sensitization [60], General toxicity [61]

**Table 6:** Prediction results for paclitaxel, trametinib, and dabrafenib.

Cell Lines	Tissue	Endpoint	Paclitaxel	Trametinib	Dabrafenib
*Non-tumor cell lines*					
GES1	Stomach	pIC_50_	7.825	4.979	4.848
HaCaT	Skin	pIC_50_	7.459	5.187	4.924
HEK293	Kidney	pIC_50_	6.699	5.329	5.269
HEK-293T	Kidney	pIC_50_	7.716	5.914	5.130
HUVEC	Endothelium	pGI_50_	8.436	5.250	5.266
HUVEC	Endothelium	pIC_50_	7.141	6.743	5.893
MCF-10A	Mammary gland	pIC_50_	3.803	5.566	5.111
MRC5	Lung	pIC_50_	9.063	4.953	5.030
PBMC	Blood	pIC_50_	5.489	6.502	7.128
TERT-RPE1	Retina	pIC_50_	5.117	5.564	5.293
WI-38	Lung	pIC_50_	7.036	5.652	4.721
Mean value			6.889	5.604	5.328
*Tumor cell lines*					
A-431	Skin	pGI_50_	8.628	6.182	5.716
A-431	Skin	pIC_50_	8.119	5.911	5.334
Caco-2	Colon	pIC_50_	7.380	5.258	5.186
COLO205	Colon	pIC_50_	8.470	7.579	8.140
HCT-8	Colon	pIC_50_	7.292	6.196	6.684
HepG2	Liver	pIC_50_	7.173	5.394	5.509
SW-620	Colon	pIC_50_	7.519	7.030	5.656
THP-1	Blood	pGI_50_	5.385	5.269	5.649
THP-1	Blood	pIC_50_	5.631	7.157	6.556
U-937	Lymphoid	pGI_50_	8.395	5.773	6.309
U-937	Lymphoid	pIC_50_	8.536	6.906	6.566
Mean value			7.502	6.241	6.119

## Data Availability

A web interface implementing the selected QSAR model is available at https://way2drug.com/clc-pred/ (accessed on 12 December 2025) for prediction using chemical structures, entering a SMILES string or searching by a common compound name.

## Supplementary Materials

The following supporting information can be downloaded at: https://www.mdpi.com/article/10.3390/ijms262412063/s1.

## Abbreviations

The following abbreviations are used in this manuscript:

ATCC	American Type Culture Collection
CLC-Pred	Cell Line Cytotoxicity Predictor (web application)
HUVEC	Human Umbilical Vein Endothelial Cells
MNA	Multilevel Neighborhoods of Atoms
OECD	Organization for Economic Cooperation and Development
PBMC	Peripheral Blood Mononuclear Cells
RBF–SCR	Radial Basis Function–Self Consistent Regression
QNA	Quantitative Neighborhoods of Atoms
QSAR	Quantitative Structure–Activity Relationship
TI	Therapeutic Index
