# Supplementary material for: QSAR Modeling for Predicting IC50 and GI50 Values for Human Cell Lines Used in Toxicological Studies
Source: Int J Mol Sci. 2025 Dec 15;26(24):12063. doi: 10.3390/ijms262412063 (PMC12733303; doi:10.3390/ijms262412063)
Supplement: Supplementary file 1 [file ijms-26-12063-s001.zip › ijms-3986078-supplementary.pdf]

Supplements

to the paper of Alexey A. Lagunin, Elena Yu. Lisitsa, Anastasia V. Rudik, Sergey M. Ivanov, Alexander V. Dmitriev, Elena S. Muraviova, Dmitry A. Filimonov, Vladimir V. Poroikov

"QSAR modeling for predicting IC_{50} and GI_{50} values for human cell lines used in toxicological studies"

Contents

Figure S1. The scatter plot of pIC_{50} values (LOO CV) of the QSAR model for BEAS-2B created by GUSAR 3

Figure S2. The scatter plot of pIC_{50} values (LOO CV) of the QSAR model for BJ created by GUSAR 3

Figure S3. The scatter plot of pIC_{50} values (LOO CV) of the QSAR model for CCD-18Co created by GUSAR 4

Figure S4. The scatter plot of pIC_{50} values (LOO CV) of the QSAR model for GES1 created by GUSAR 4

Figure S5. The scatter plot of pIC_{50} values (LOO CV) of the QSAR model for HaCaT created by GUSAR 5

Figure S6. The scatter plot of pIC_{50} values (LOO CV) of the QSAR model for HEK-293 created by GUSAR 5

Figure S7. The scatter plot of pIC_{50} values (LOO CV) of the QSAR model for HEK-293T created by GUSAR 6

Figure S8. The scatter plot of pIC_{50} values (LOO CV) of the QSAR model for HFF created by GUSAR 6

Figure S9. The scatter plot of pIC_{50} values (LOO CV) of the QSAR model for HFL1 created by GUSAR 7

Figure S10. The scatter plot of pIC_{50} values (LOO CV) of the QSAR model for HMEC-1 created by GUSAR 7

Figure S11. The scatter plot of pIC_{50} values (LOO CV) of the QSAR model for HUVEC created by GUSAR 8

Figure S12. The scatter plot of pIC_{50} values (LOO CV) of the QSAR model for MCF-10A created by GUSAR 8

Figure S13. The scatter plot of pIC_{50} values (LOO CV) of the QSAR model for MRC5 created by GUSAR 9

Figure S14. The scatter plot of pIC_{50} values (LOO CV) of the QSAR model for NHDF created by GUSAR 9

Figure S15. The scatter plot of pIC_{50} values (LOO CV) of the QSAR model for PBMC created by GUSAR 10

Figure S16. The scatter plot of pIC_{50} values (LOO CV) of the QSAR model for TERT-RPE1 created by GUSAR 10

Figure S17. The scatter plot of pIC_{50} values (LOO CV) of the QSAR model for WI-38 created by GUSAR 11

Figure S18. The scatter plot of pGI_{50} values (LOO CV) of the QSAR model for HUVEC created by GUSAR 11

Figure S19. The scatter plot of pIC_{50} values (LOO CV) of the QSAR model for A-375 created by GUSAR 12

Figure S20. The scatter plot of pIC_{50} values (LOO CV) of the QSAR model for A-431 created by GUSAR 12

Figure S21. The scatter plot of pIC_{50} values (LOO CV) of the QSAR model for Caco-2 created by GUSAR 13

Figure S22. The scatter plot of pIC_{50} values (LOO CV) of the QSAR model for Caki-1 created by GUSAR 13

Figure S23. The scatter plot of pIC_{50} values (LOO CV) of the QSAR model for Calu-1created by GUSAR 14

Figure S24. The scatter plot of pIC_{50} values (LOO CV) of the QSAR model for COLO 205 created by GUSAR 14

Figure S25. The scatter plot of pIC_{50} values (LOO CV) of the QSAR model for HCT-8 created by GUSAR 15

Figure S26. The scatter plot of pIC_{50} values (LOO CV) of the QSAR model for HepG2 created by GUSAR 15

Figure S27. The scatter plot of pIC_{50} values (LOO CV) of the QSAR model for SH-SY5Y created by GUSAR 16

Figure S28. The scatter plot of pIC_{50} values (LOO CV) of the QSAR model for SW-620 created by GUSAR 16

Figure S29. The scatter plot of pIC_{50} values (LOO CV) of the QSAR model for THP-1 created by GUSAR 17

Figure S30. The scatter plot of pIC_{50} values (LOO CV) of the QSAR model for U-937 created by GUSAR 17

Figure S31. The scatter plot of pGI_{50} values (LOO CV) of the QSAR model for A-431 created by GUSAR 18

Figure S32. The scatter plot of pGI_{50} values (LOO CV) of the QSAR model for COLO 205 created by GUSAR 18

Figure S33. The scatter plot of pGI_{50} values (LOO CV) of the QSAR model for HepG2 created by GUSAR 19

Figure S34. The scatter plot of pGI_{50} values (LOO CV) of the QSAR model for THP-1 created by GUSAR 19

Figure S35. The scatter plot of pGI_{50} values (LOO CV) of the QSAR model for U-937 created by GUSAR 20

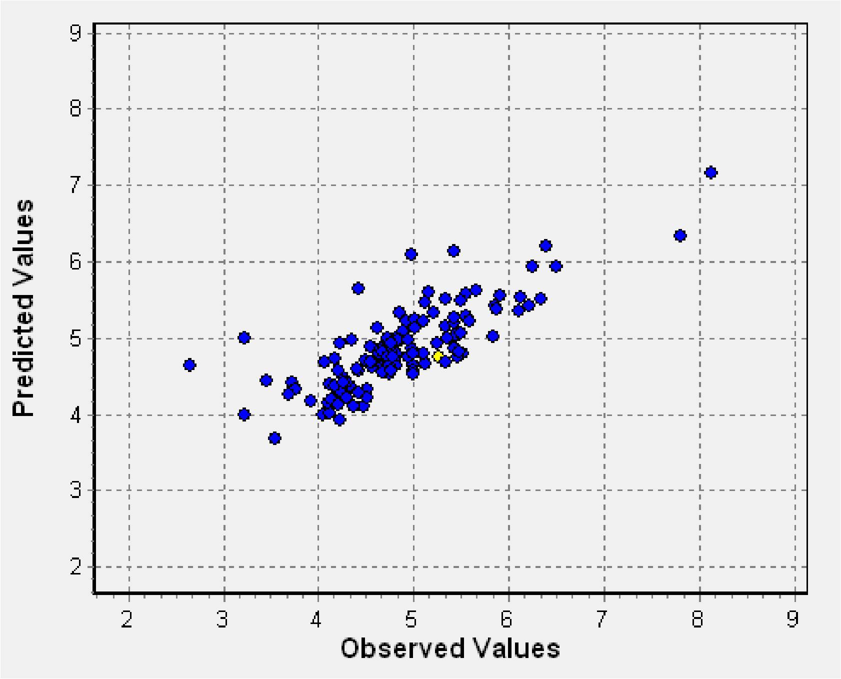

Figure S1. The scatter plot of pIC_{50} values (LOO CV) of the QSAR model for BEAS-2B created by GUSAR 2

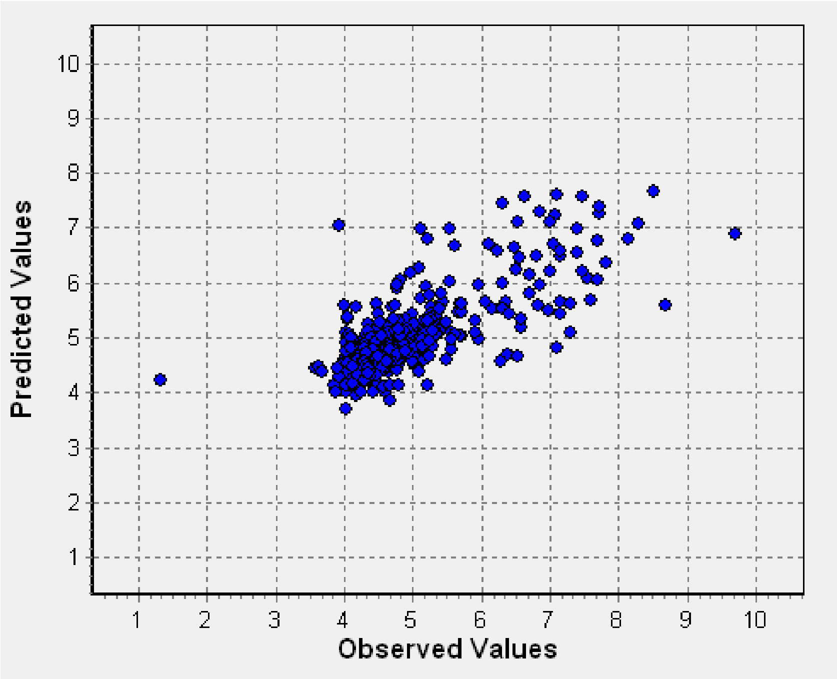

Figure S2. The scatter plot of pIC_{50} values (LOO CV) of the QSAR model for BJ created by GUSAR

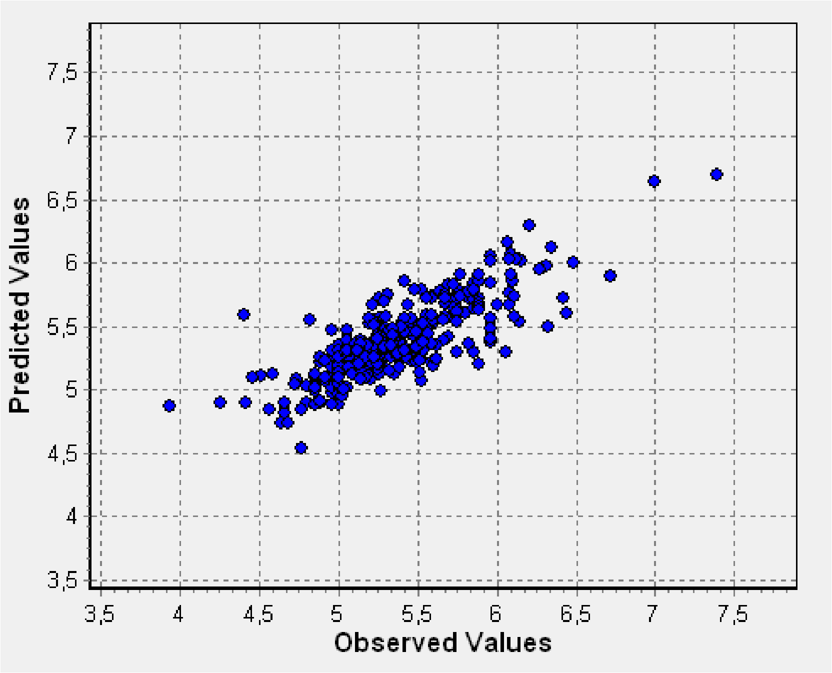

Figure S3. The scatter plot of pIC_{50} values (LOO CV) of the QSAR model for CCD-18Co created by GUSAR

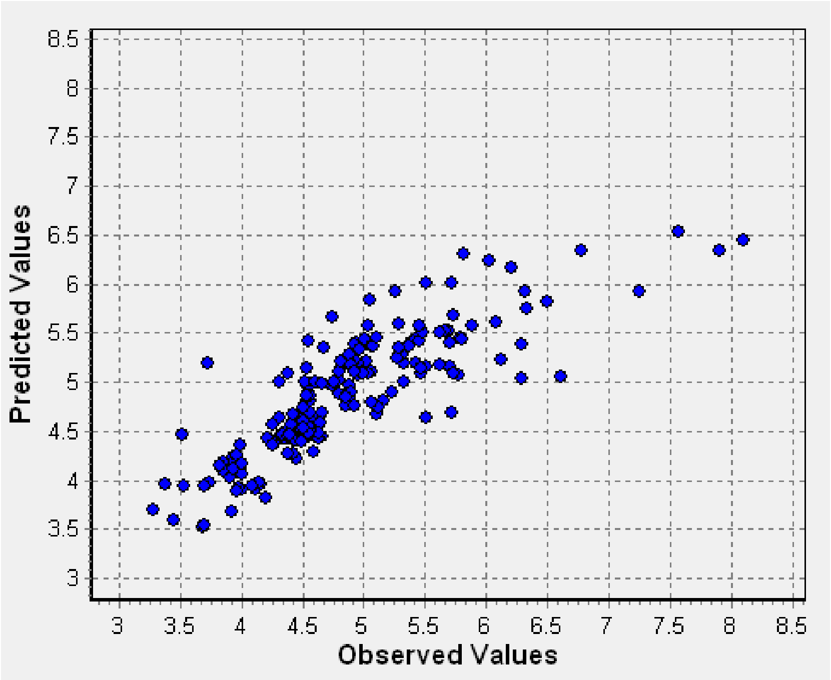

Figure S4. The scatter plot of pIC_{50} values (LOO CV) of the created QSAR model for GES1 from GUSAR.

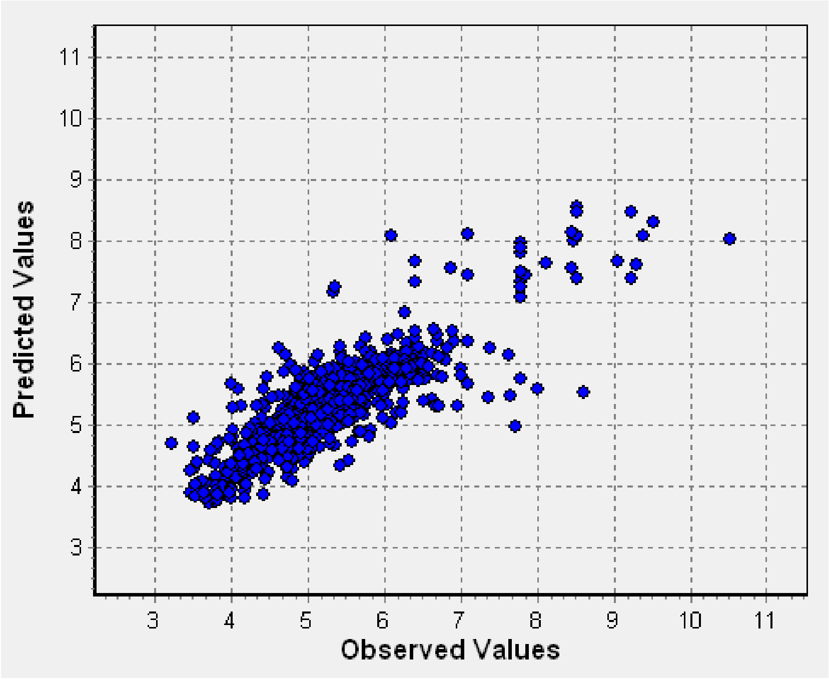

Figure S5. The scatter plot of pIC_{50} values (LOO CV) of the QSAR model for HaCaT created by GUSAR.

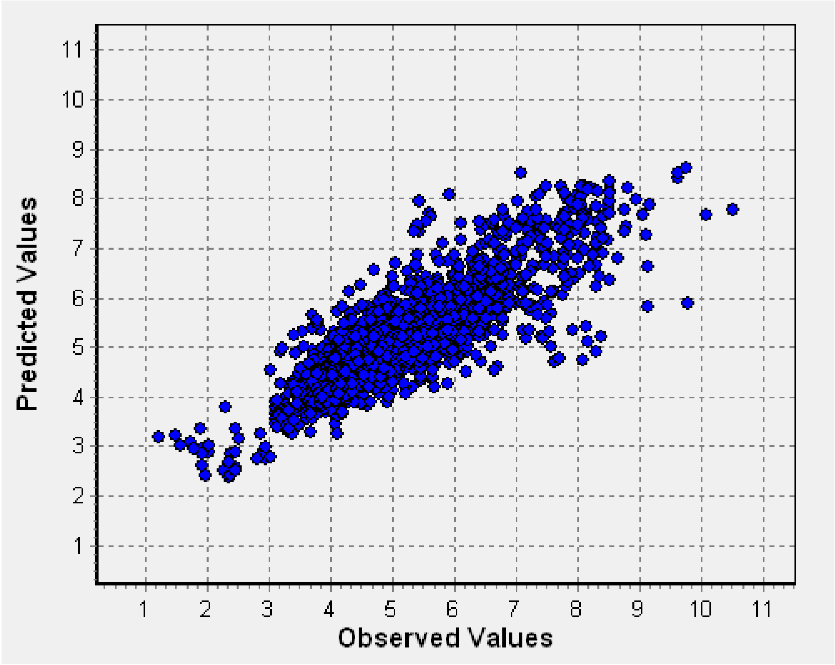

Figure S6. The scatter plot of pIC_{50} values (LOO CV) of the QSAR model for HEK-293 created by GUSAR.

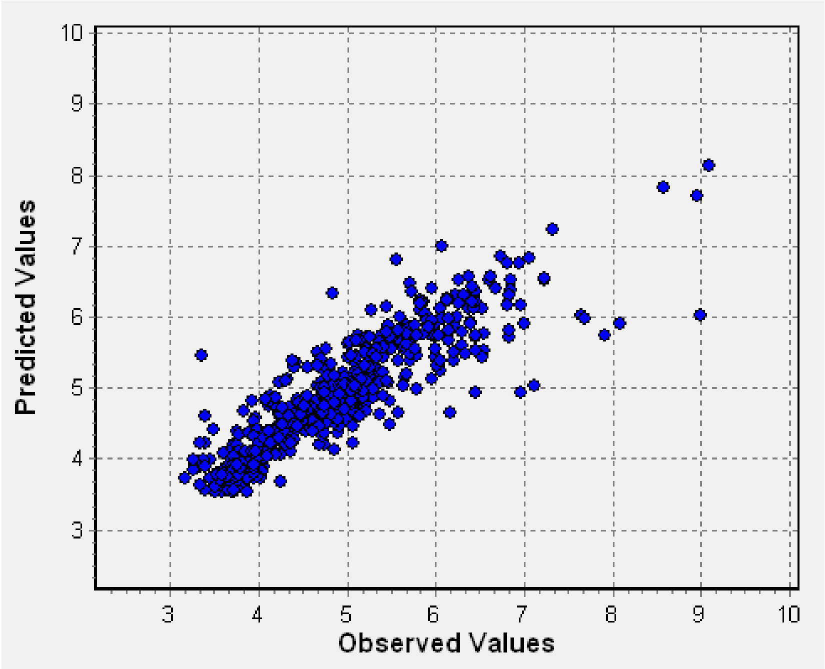

Figure S7. The scatter plot of pIC_{50} values (LOO CV) of the QSAR model for HEK-293T created by GUSAR.

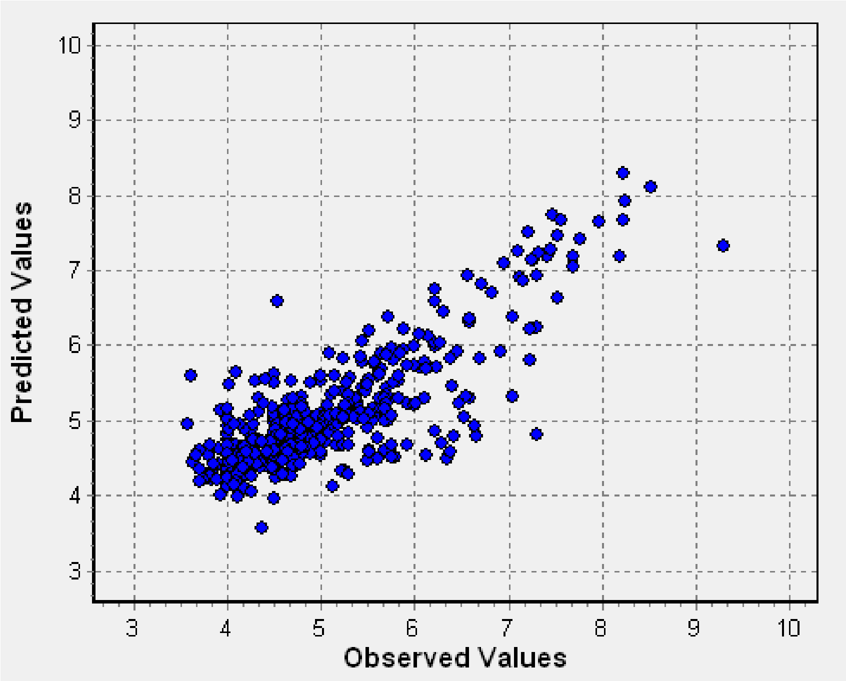

Figure S8. The scatter plot of pIC_{50} values (LOO CV) of the QSAR model for HFF created by GUSAR

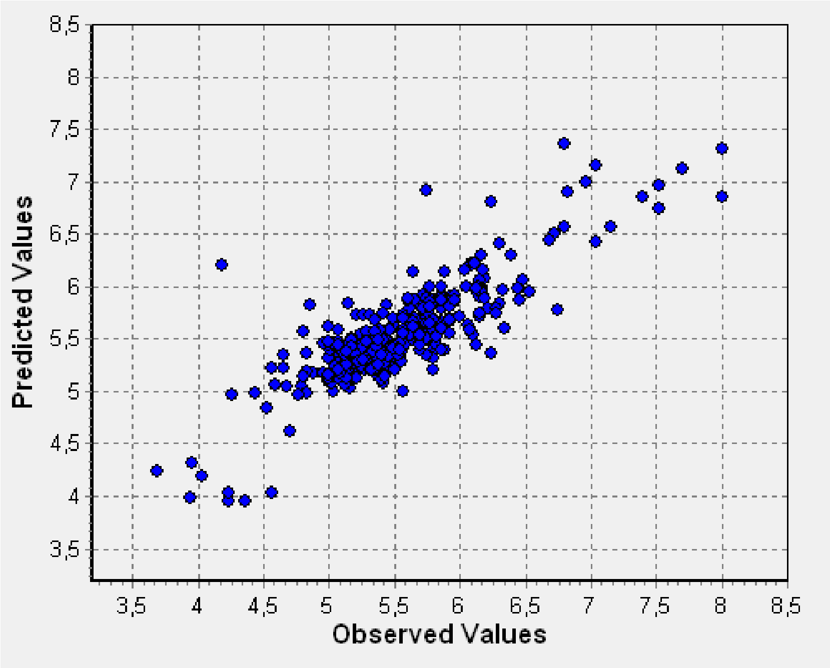

Figure S9. The scatter plot of pIC_{50} values (LOO CV) of the QSAR model for HFL1 created by GUSAR

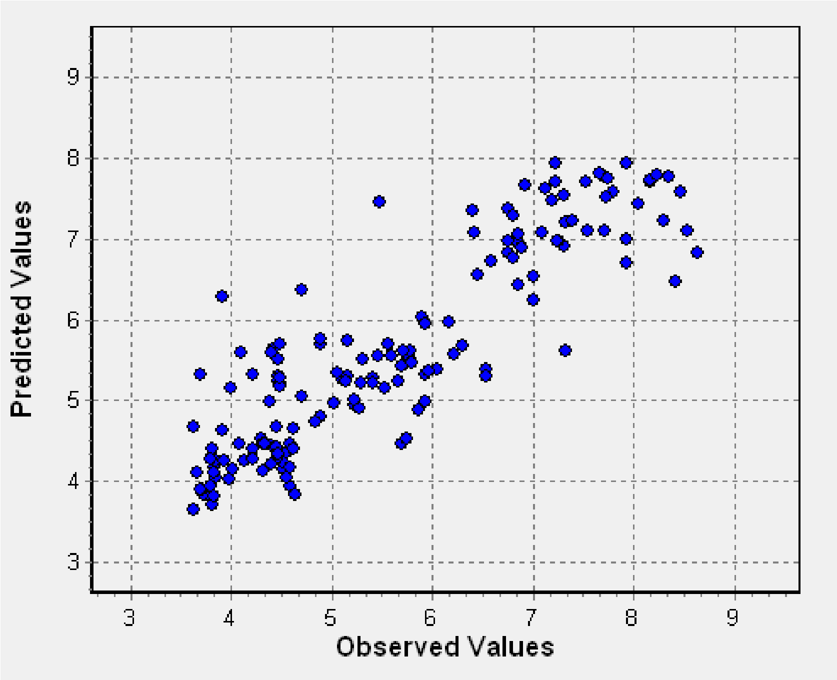

Figure S10. The scatter plot of pIC_{50} values (LOO CV) of the QSAR model for HMEC-1 created by GUSAR

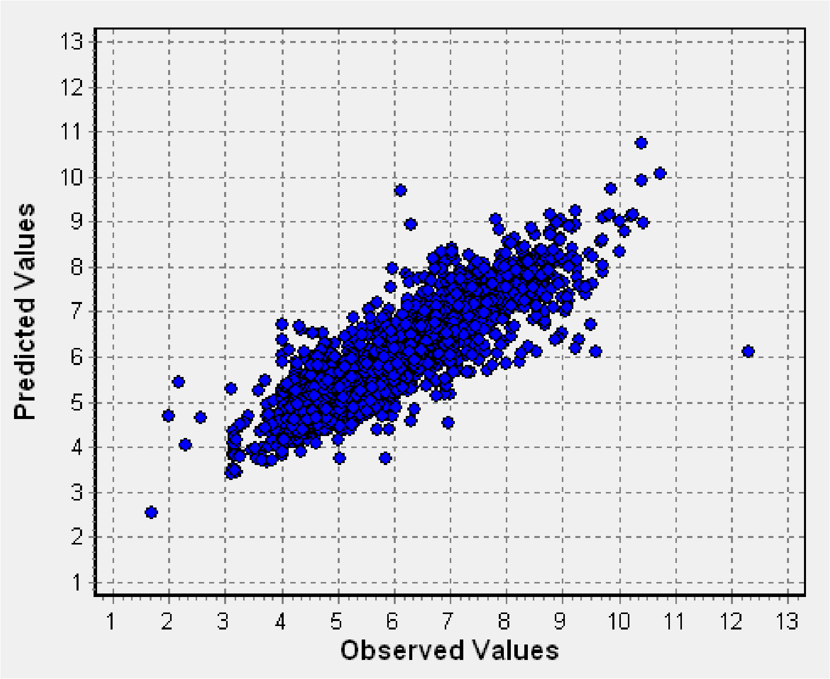

Figure S11. The scatter plot of pIC_{50} values (LOO CV) of the QSAR model for HUVEC created by GUSAR.

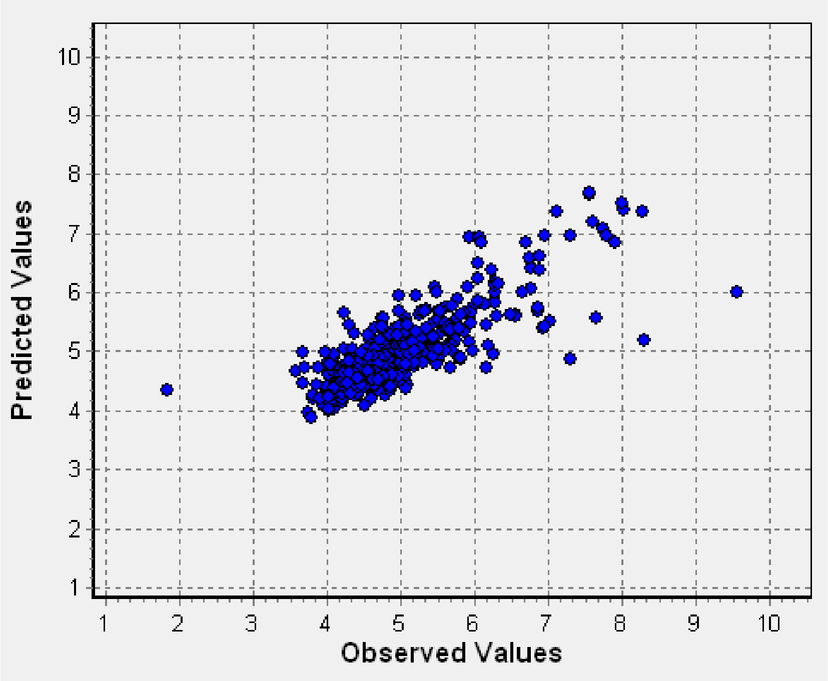

Figure S12. The scatter plot of pIC_{50} values (LOO CV) of the QSAR model for MCF-10A created by GUSAR.

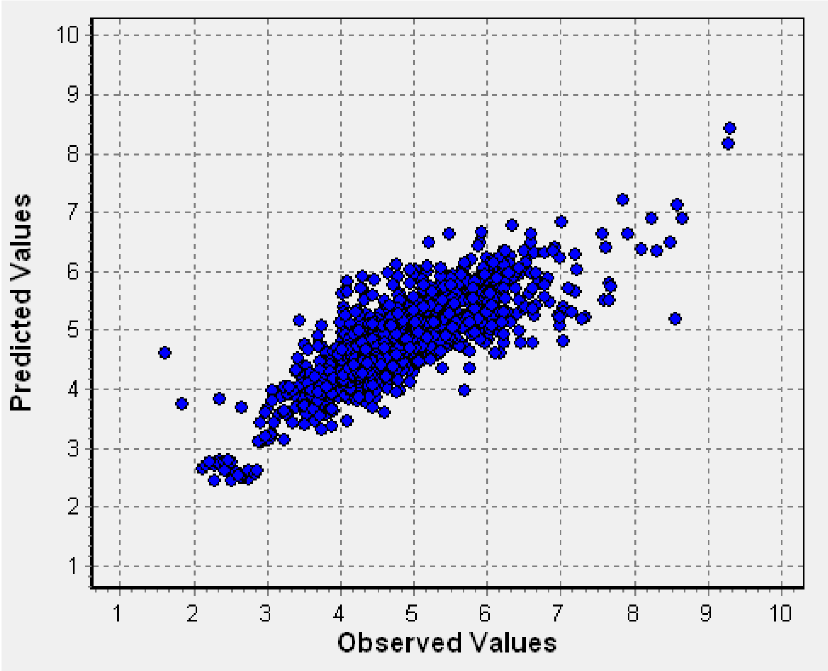

Figure S13. The scatter plot of pIC_{50} values (LOO CV) of the QSAR model for MRC5 created by GUSAR.

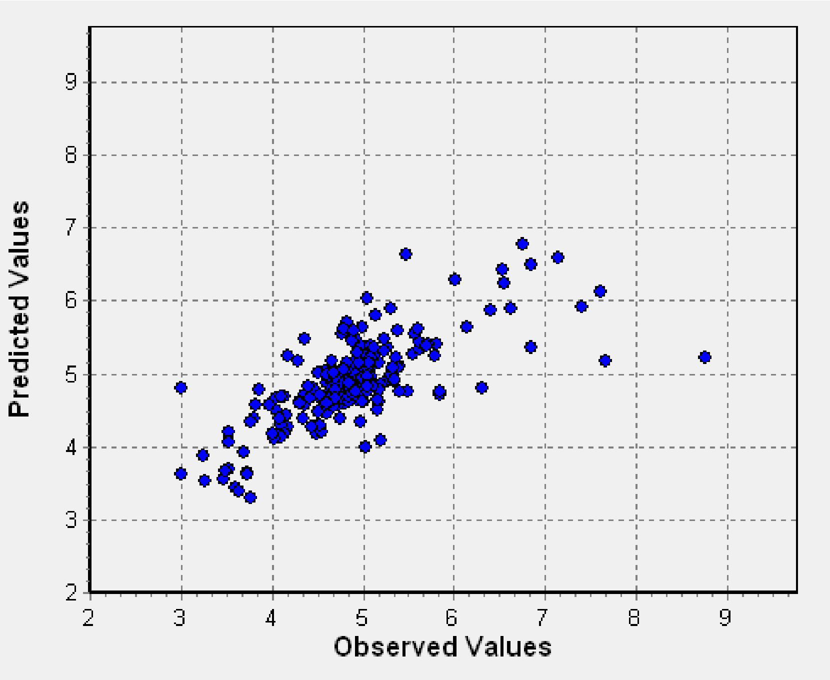

Figure S14. The scatter plot of pIC_{50} values (LOO CV) of the QSAR model for NHDF created by GUSAR

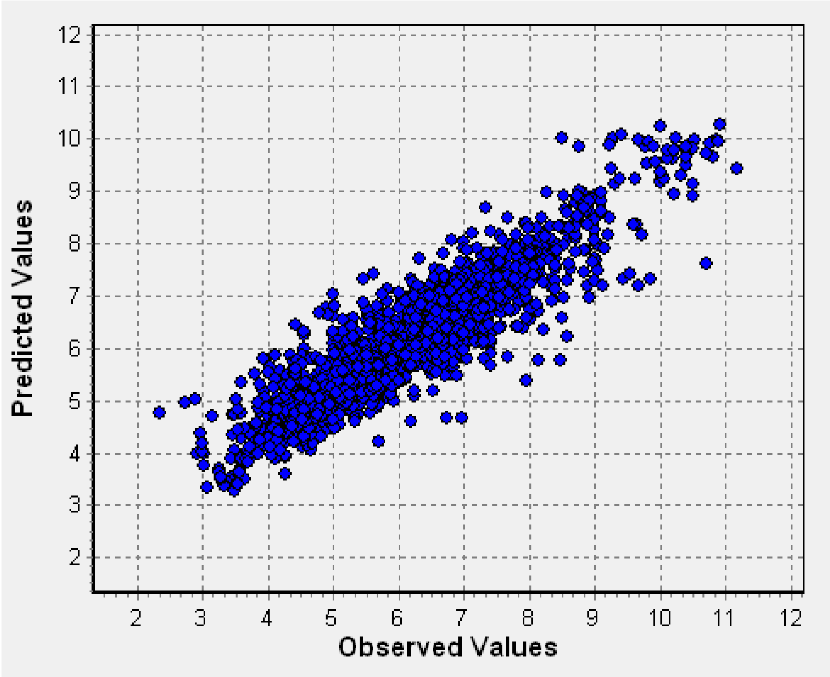

Figure S15. The scatter plot of pIC_{50} values (LOO CV) of the QSAR model for PBMC created by GUSAR.

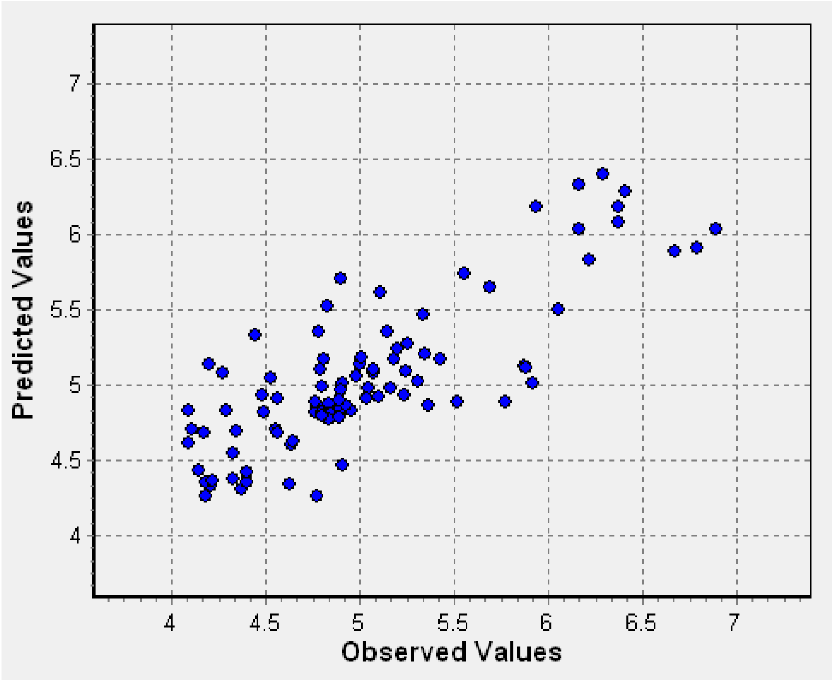

Figure S16. The scatter plot of pIC_{50} values (LOO CV) of the QSAR model for TERT-RPE1 created by GUSAR

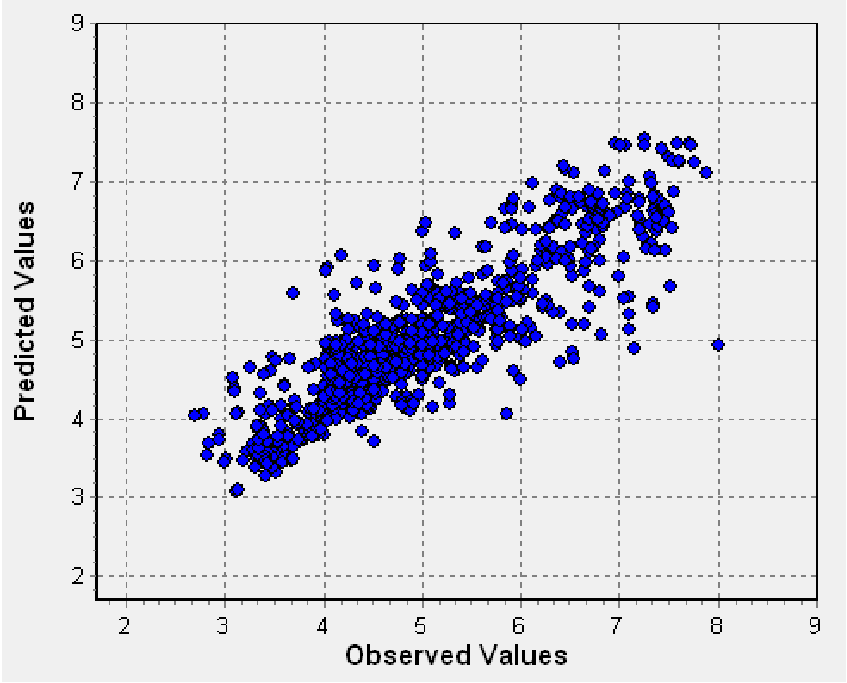

Figure S17. The scatter plot of pIC_{50} values (LOO CV) of the QSAR model for WI-38 created by GUSAR

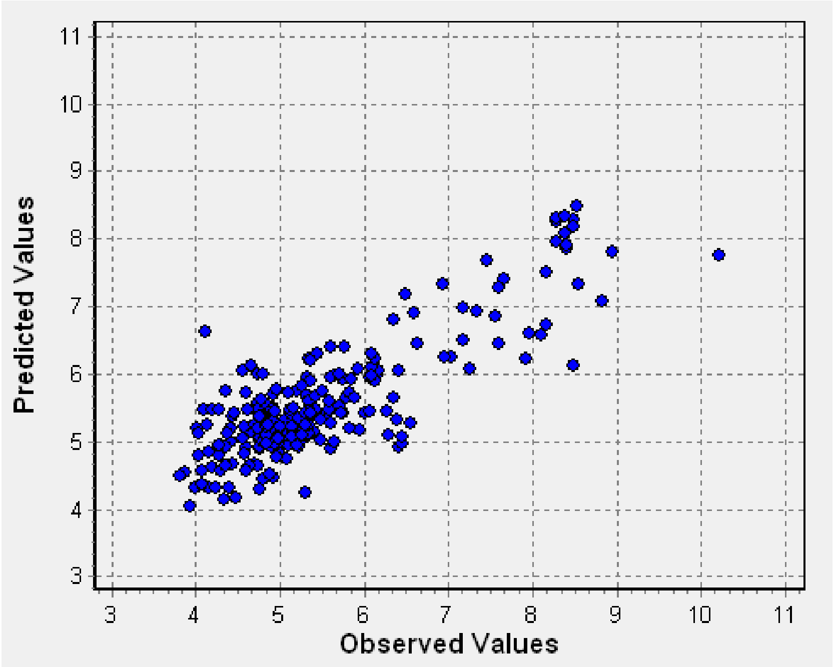

Figure S18. The scatter plot of pGI_{50} values (LOO CV) of the QSAR model for HUVEC created by GUSAR

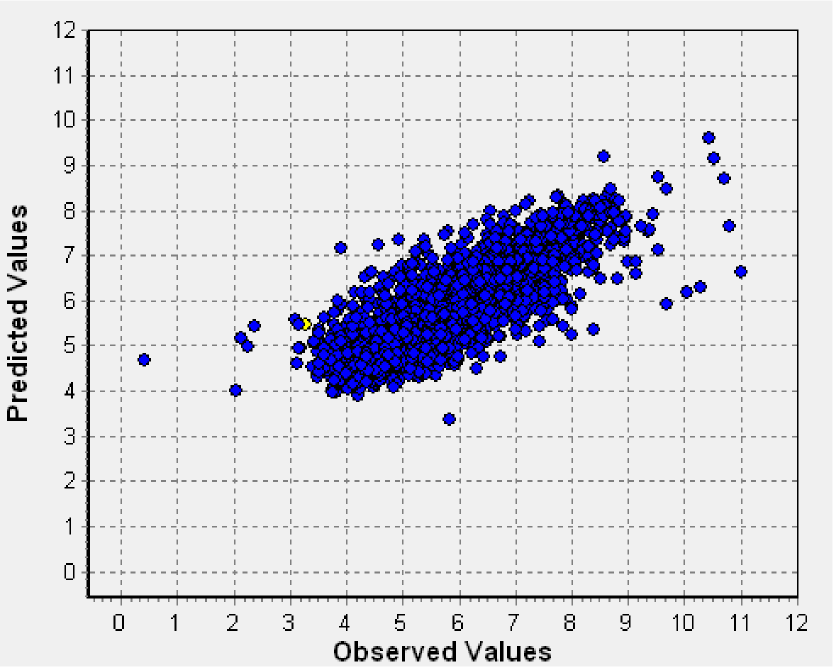

Figure S19. The scatter plot of pIC_{50} values (LOO CV) of the QSAR model for A-375 created by GUSAR

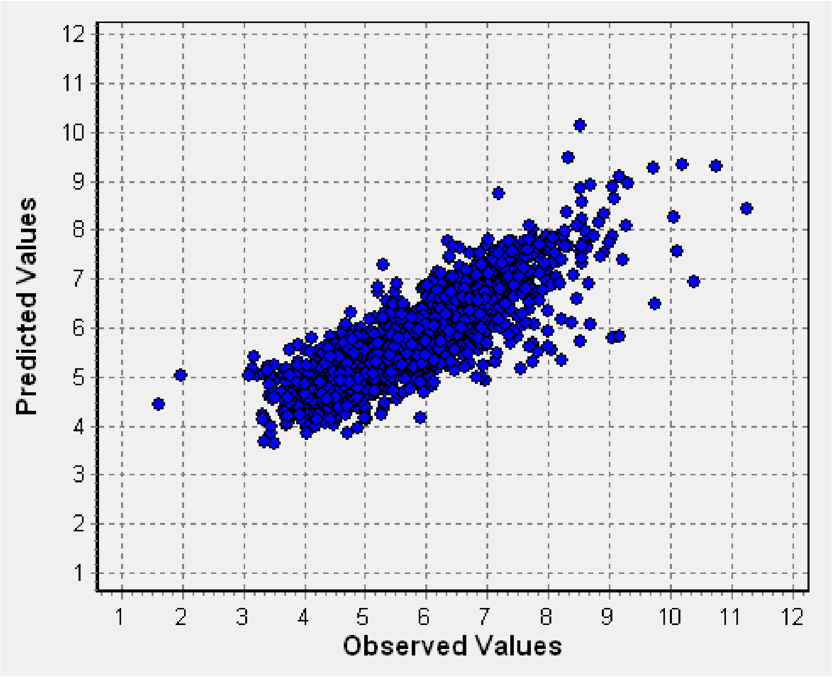

Figure S20. The scatter plot of pIC_{50} values (LOO CV) of the QSAR model for A-431 created by GUSAR

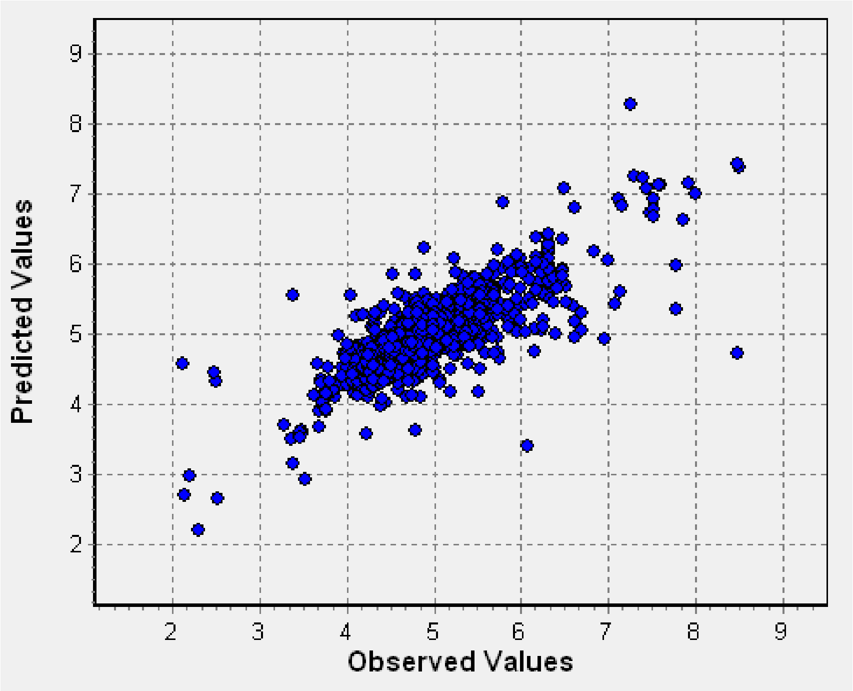

Figure S21. The scatter plot of pIC_{50} values (LOO CV) of the QSAR model for Caco-2 created by GUSAR

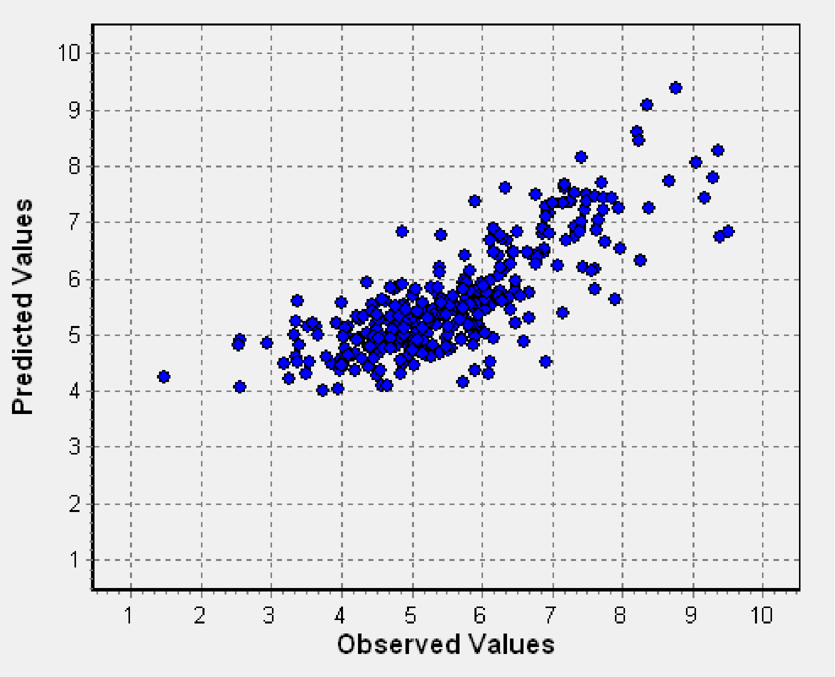

Figure S22. The scatter plot of pIC_{50} values (LOO CV) of the QSAR model for Caki-1 created by GUSAR

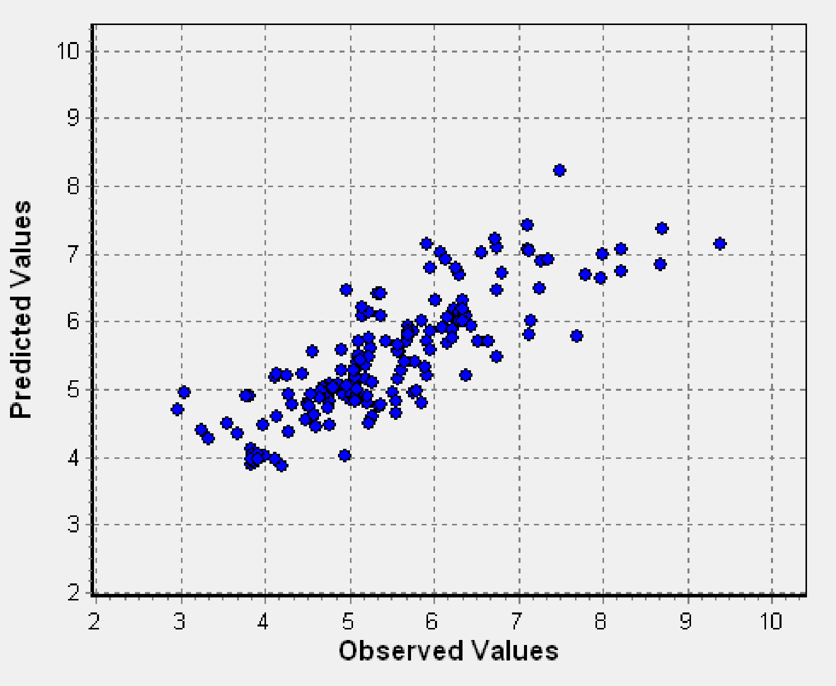

Figure S23. The scatter plot of pIC_{50} values (LOO CV) of the QSAR model for Calu-1created by GUSAR

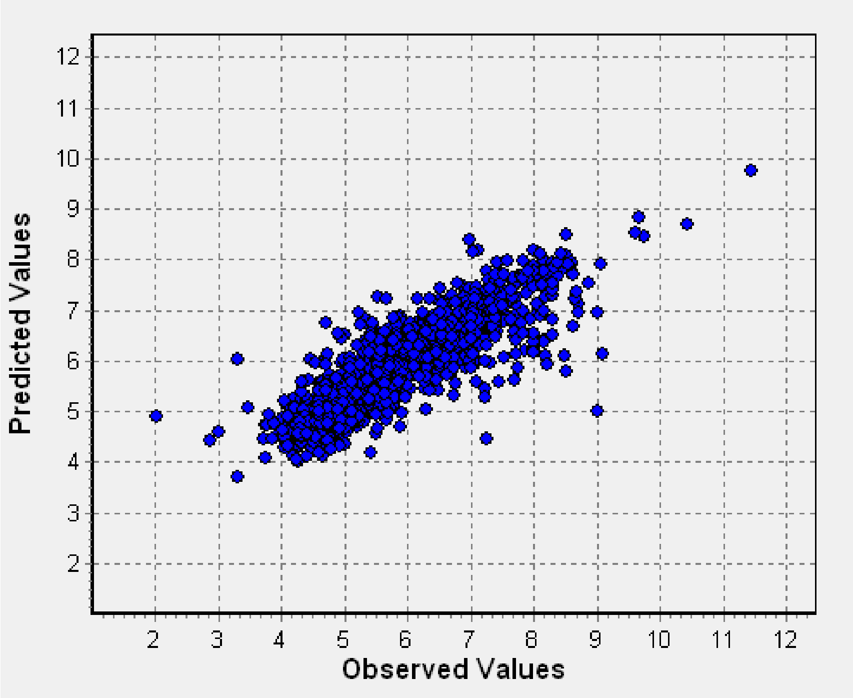

Figure S24. The scatter plot of pIC_{50} values (LOO CV) of the QSAR model for COLO 205 created by GUSAR

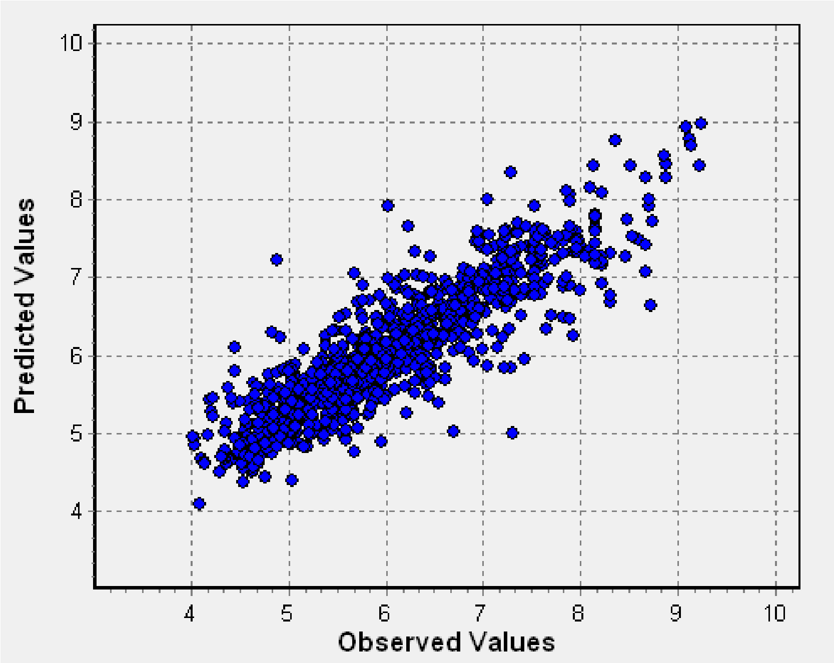

Figure S25. The scatter plot of pIC_{50} values (LOO CV) of the QSAR model for HCT-8 created by GUSAR

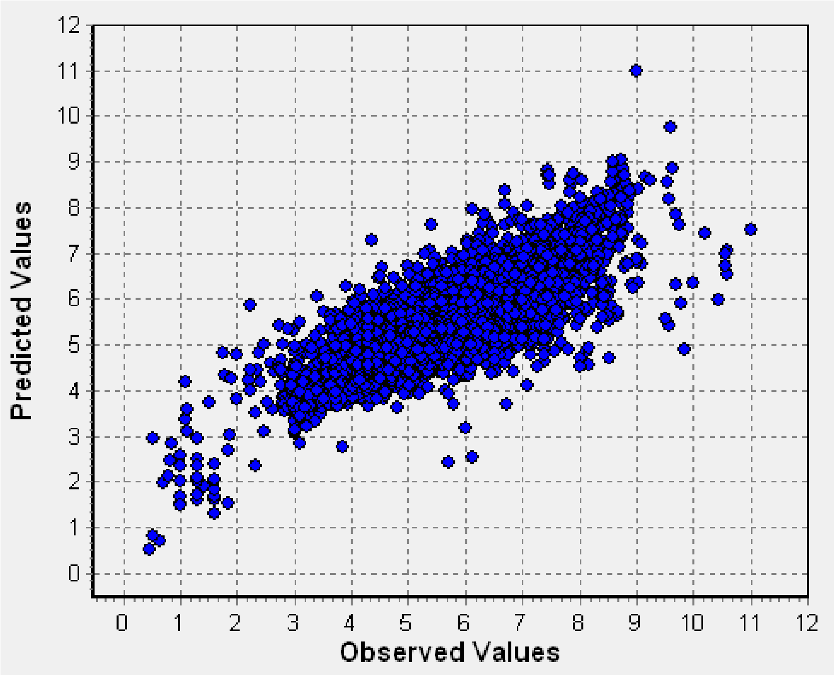

Figure S26. The scatter plot of pIC_{50} values (LOO CV) of the QSAR model for HepG2 created by GUSAR

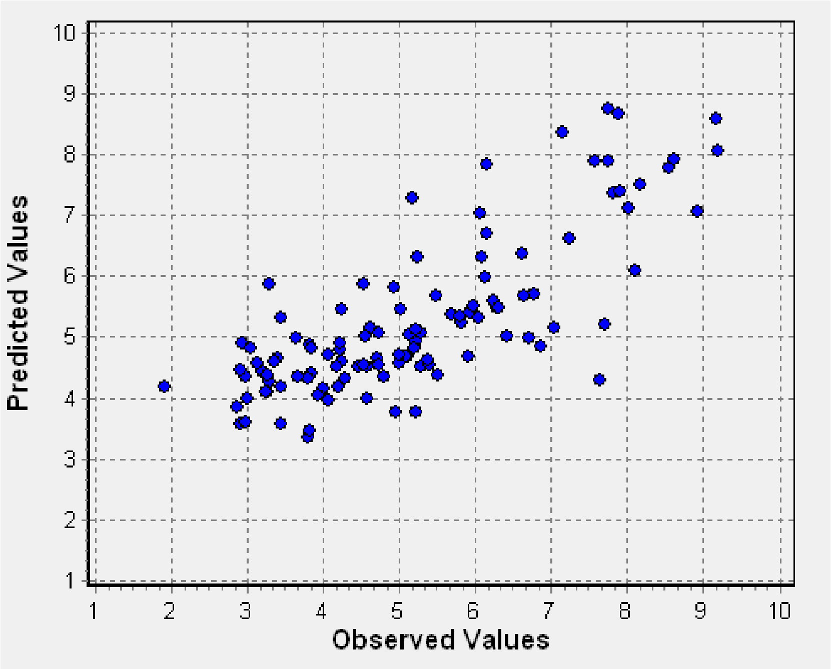

Figure S27. The scatter plot of pIC_{50} values (LOO CV) of the QSAR model for SH-SY5Y created by GUSAR

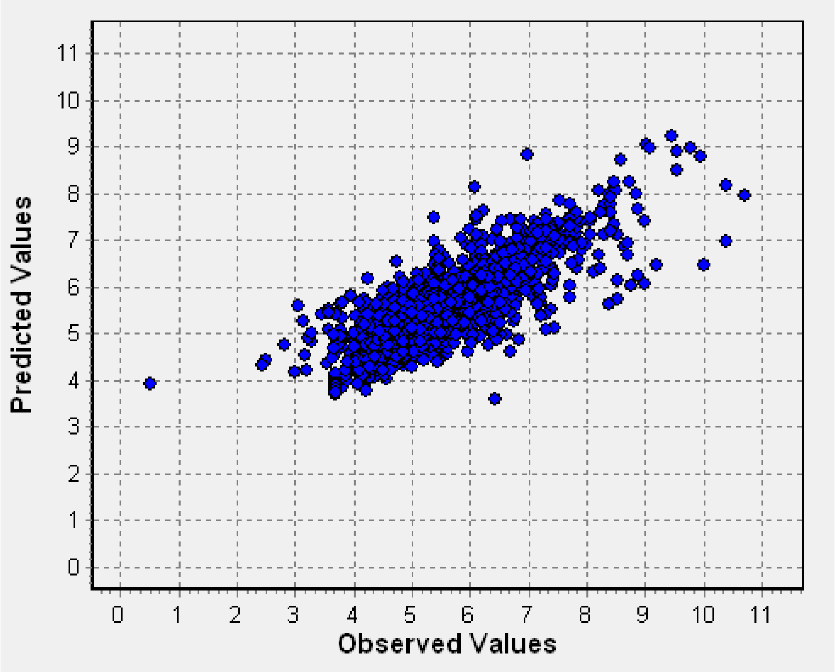

Figure S28. The scatter plot of pIC_{50} values (LOO CV) of the QSAR model for SW-620 created by GUSAR

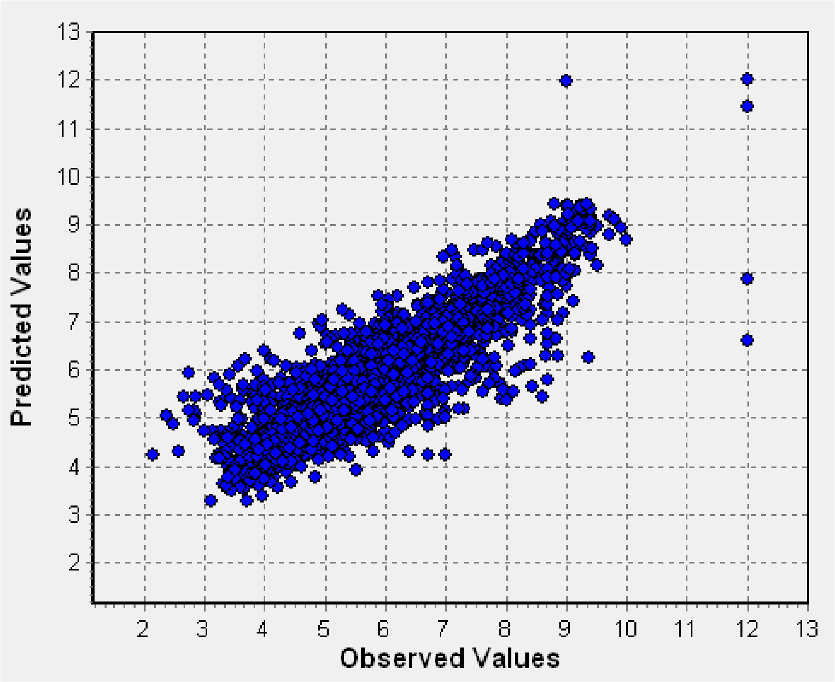

Figure S29. The scatter plot of pIC_{50} values (LOO CV) of the QSAR model for THP-1 created by GUSAR

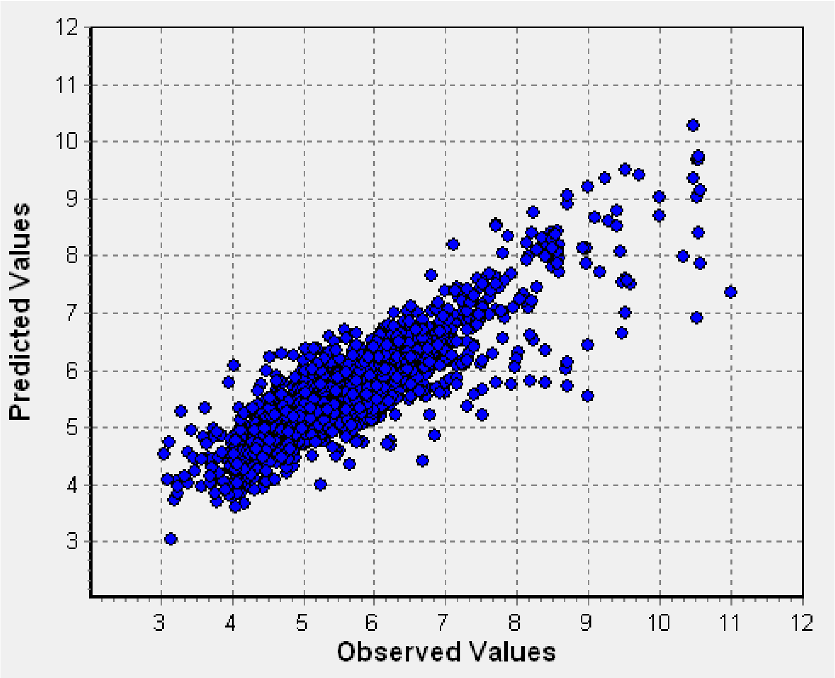

Figure S30. The scatter plot of pIC_{50} values (LOO CV) of the QSAR model for U-937 created by GUSAR

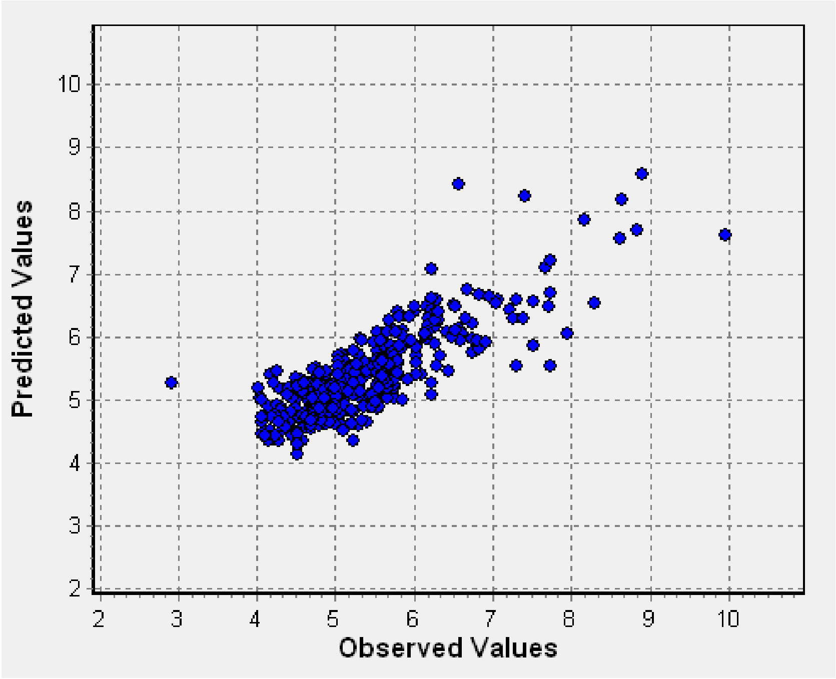

Figure S31. The scatter plot of pGI$_{50}$ values (LOO CV) of the QSAR model for A-431 created by GUSAR

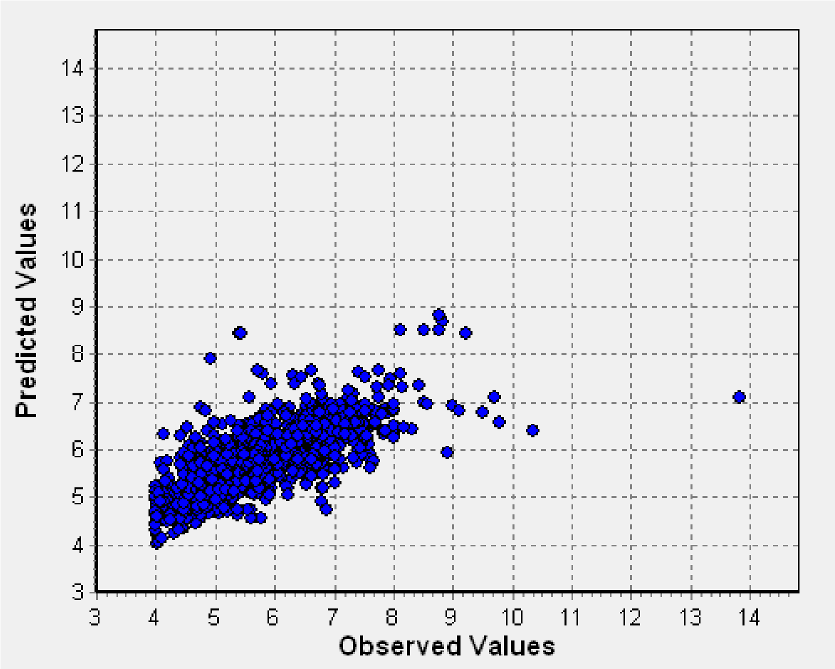

Figure S32. The scatter plot of pGI$_{50}$ values (LOO CV) of the QSAR model for COLO 205 created by GUSAR

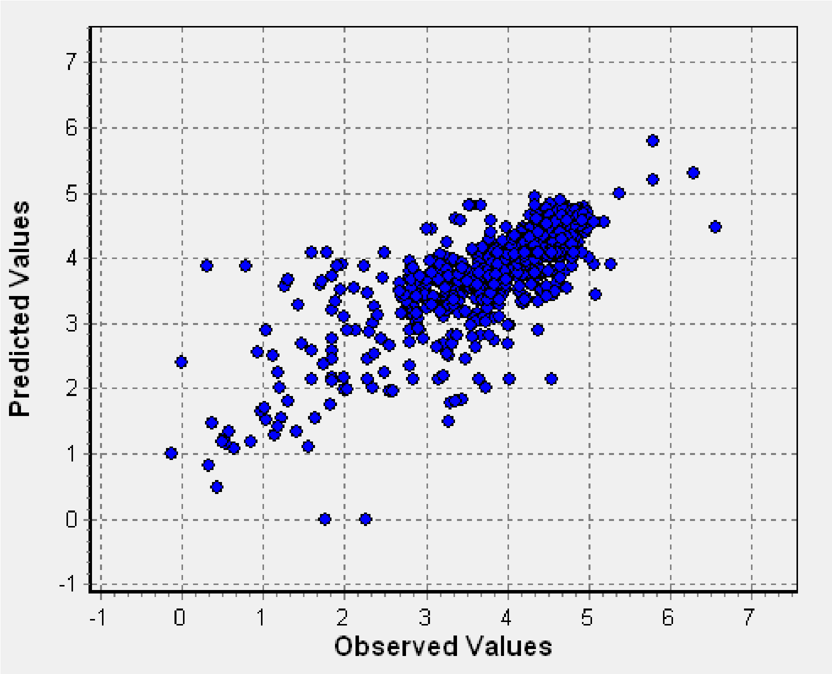

Figure S33. The scatter plot of pGI_{50} values (LOO CV) of the QSAR model for HepG2 created by GUSAR

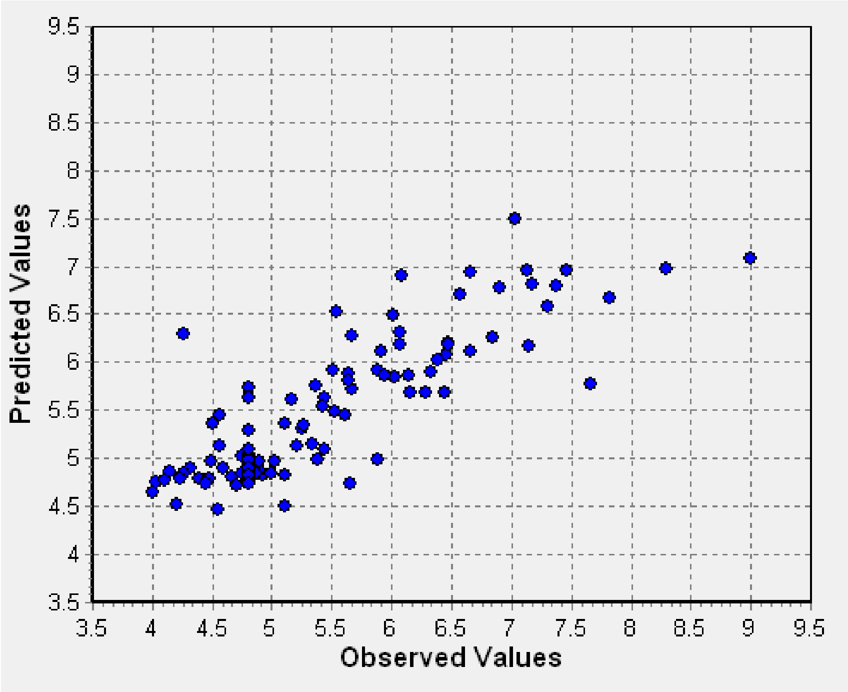

Figure S34. The scatter plot of pGI_{50} values (LOO CV) of the QSAR model for THP-1 created by GUSAR

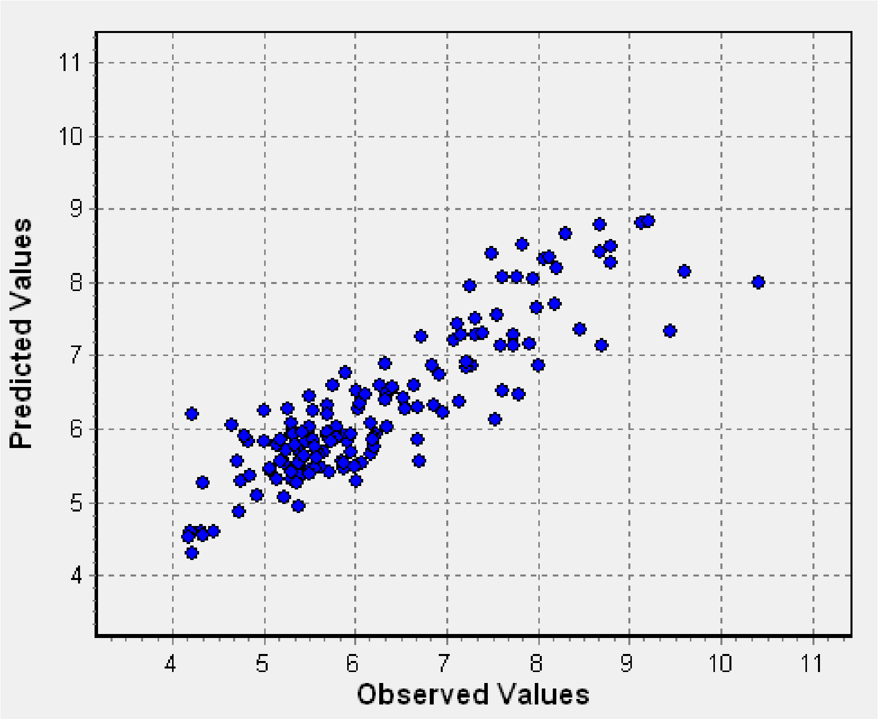

Figure S35. The scatter plot of pGI_{50} values (LOO CV) of the QSAR model for U-937 created by GUSAR
